# Developing and Validating a Multivariable Prognostic-Predictive Classifier for Treatment Escalation of Oropharyngeal Squamous Cell Carcinoma: The PREDICTR-OPC Study

**DOI:** 10.1158/1078-0432.CCR-23-1013

**Published:** 2023-10-23

**Authors:** Hisham Mehanna, Davy Rapozo, Sandra V. von Zeidler, Kevin J. Harrington, Stuart C. Winter, Andrew Hartley, Paul Nankivell, Andrew G. Schache, Philip Sloan, Edward W. Odell, Selvam Thavaraj, Keith D. Hunter, Ketan A. Shah, Gareth J. Thomas, Anna Long, Rasoul Amel-Kashipaz, Rachel M. Brown, Brendan Conn, Gillian L. Hall, Paul Matthews, Justin Weir, Yen Yeo, Miranda Pring, Catharine M.L. West, James McCaul, Pawel Golusinski, Alice Sitch, Rachel Spruce, Nikolaos Batis, Jennifer L. Bryant, Jill M. Brooks, Terence M. Jones, Francesca Buffa, Syed Haider, Max Robinson

**Affiliations:** 1 Institute of Head and Neck Studies and Education, University of Birmingham, Birmingham, United Kingdom.; 2 National Cancer Institute of Brazil, Rio de Janeiro, Brazil.; 3 Pathology Department and Biotechnology Post-graduation Program, Federal University of Espírito Santo, Vitória, Brazil.; 4 The Royal Marsden/The Institute of Cancer Research National Institute of Health Research Biomedical Research Centre, London, United Kingdom.; 5 Department of ENT-Head and Neck Surgery, Churchill Hospital, Nuffield Department of Surgery, University of Oxford, Oxford, United Kingdom.; 6 Hall-Edwards Radiotherapy Research Group, University Hospitals Birmingham, Birmingham, United Kingdom.; 7 Northwest Cancer Research Centre, Department of Molecular & Clinical Cancer Medicine, University of Liverpool Head & Neck Unit, University Hospital Aintree, Liverpool, United Kingdom.; 8 Center for Oral Health Research, Newcastle University, Newcastle upon Tyne, United Kingdom.; 9 Head and Neck Pathology, King's College London, Guy's Hospital, London, United Kingdom.; 10 Faculty of Dental, Oral and Craniofacial Sciences, King's College London, London, United Kingdom.; 11 Head and Neck Pathology at Guy's & St Thomas' Hospital NHS Foundation Trust, London, United Kingdom.; 12 Liverpool Head and Neck Centre, Molecular and Clinical Medicine, University of Liverpool, Liverpool, United Kingdom.; 13 Department of Cellular Pathology, John Radcliffe Hospital, Oxford, United Kingdom.; 14 Cancer Sciences Unit, University of Southampton, University Road, Southampton, United Kingdom.; 15 Cellular Pathology, Newcastle upon Tyne Hospitals NHS Foundation Trust, Newcastle upon Tyne, United Kingdom.; 16 University Hospitals Birmingham, Edgbaston, Birmingham, United Kingdom.; 17 Royal Infirmary of Edinburgh, Edinburgh, Scotland.; 18 Guy’s Hospital, London, United Kingdom.; 19 Department of Pathology, University Hospitals Coventry and Warwickshire, United Kingdom.; 20 Department of Cellular Pathology, Charing Cross Hospital, Imperial College Healthcare Trust, London, United Kingdom.; 21 Department of Pathology and Laboratory Medicine, KK Women's and Children's Hospital, Singapore.; 22 Bristol Dental School, University of Bristol, Bristol, United Kingdom.; 23 Division of Cancer Studies, University of Manchester, Christie Hospital NHS Trust, Manchester Academic Health Science Centre, Manchester, United Kingdom.; 24 Department of Maxillofacial and Head and Neck Surgery, Queen Elizabeth II Hospital, Glasgow, Scotland.; 25 Department of Otolaryngology and Maxillofacial Surgery, University of Zielona Gora, Zielona Gora, Poland.; 26 Institute of Applied Health Research, University of Birmingham, Birmingham, United Kingdom.; 27 NIHR Birmingham Biomedical Research Centre, University Hospitals Birmingham NHS Foundation Trust, Birmingham, United Kingdom.; 28 University of Birmingham, United Kingdom.; 29 Northwest Cancer Research Centre, Department of Molecular & Clinical Cancer Medicine, University of Liverpool Head & Neck Unit, University Hospital Aintree, Liverpool, United Kingdom.; 30 Department of Oncology, University of Oxford, Oxford, United Kingdom.; 31 Department of Computing Sciences, Bocconi University, Milano, Italy.; 32 The Breast Cancer Now Toby Robins Research Centre, The Institute of Cancer Research, London, United Kingdom.

## Abstract

**Purpose:**

While there are several prognostic classifiers, to date, there are no validated predictive models that inform treatment selection for oropharyngeal squamous cell carcinoma (OPSCC).

Our aim was to develop clinical and/or biomarker predictive models for patient outcome and treatment escalation for OPSCC.

**Experimental Design:**

We retrospectively collated clinical data and samples from a consecutive cohort of OPSCC cases treated with curative intent at ten secondary care centers in United Kingdom and Poland between 1999 and 2012. We constructed tissue microarrays, which were stained and scored for 10 biomarkers. We then undertook multivariable regression of eight clinical parameters and 10 biomarkers on a development cohort of 600 patients. Models were validated on an independent, retrospectively collected, 385-patient cohort.

**Results:**

A total of 985 subjects (median follow-up 5.03 years, range: 4.73–5.21 years) were included. The final biomarker classifier, comprising p16 and survivin immunohistochemistry, high-risk human papillomavirus (HPV) DNA *in situ* hybridization, and tumor-infiltrating lymphocytes, predicted benefit from combined surgery + adjuvant chemo/radiotherapy over primary chemoradiotherapy in the high-risk group [3-year overall survival (OS) 63.1% vs. 41.1%, respectively, HR = 0.32; 95% confidence interval (CI), 0.16–0.65; *P* = 0.002], but not in the low-risk group (HR = 0.4; 95% CI, 0.14–1.24; *P* = 0.114). On further adjustment by propensity scores, the adjusted HR in the high-risk group was 0.34, 95% CI = 0.17–0.67, *P* = 0.002, and in the low-risk group HR was 0.5, 95% CI = 0.1–2.38, *P* = 0.384. The concordance index was 0.73.

**Conclusions:**

We have developed a prognostic classifier, which also appears to demonstrate moderate predictive ability. External validation in a prospective setting is now underway to confirm this and prepare for clinical adoption.

**Translational Relevance**

There are currently two different modalities, chemoradiotherapy or surgery with adjuvant therapy, for treatment for head and neck cancer, with different toxicity profiles. There are no validated predictors of treatment response in routine clinical use. Consequently, treatment selection is mainly based on burden of disease (TNM staging) and clinician and patient preference. We developed a prognostic classifier that may have predictive properties to help select treatment based on tumor biology and validated it on an independent cohort. The classifier, comprising p16 and survivin immunohistochemistry, high-risk human papillomavirus (HPV) DNA *in situ* hybridization, and tumor-infiltrating lymphocytes, predicts which patients with oropharyngeal cancer benefit more from treatment escalation by combining surgery with chemotherapy. Further external validation on a prospective cohort is indicated for clinical implementation.

## Introduction

Over the past two decades, there has been a remarkable change in the epidemiology and aetiology of oropharyngeal squamous cell carcinoma (OPSCC; ref. 1), which is now one of the most rapidly rising cancers in the Western world (2–4). This increasing incidence has been attributed to oncogenic human papillomavirus (HPV) infection (5, 6). Patients with HPV-related OPSCC demonstrate better survival outcomes than those with HPV-negative cancers at the same site (7, 8).

For many years, the standard treatment for advanced OPSCC was chemoradiotherapy (CRT). However, with the advent of transoral laser microsurgery (TLM), and more recently, transoral robotic surgery (TORS), surgery followed by adjuvant treatment has gained popularity. In this paradigm, approximately 40% to 50% of patients receive triple therapy with surgery, radiotherapy, and chemotherapy, and most of the remainder receive surgery and radiotherapy (9, 10). Currently, selection between these paradigms is usually guided by clinical judgment on surgical resectability, clinician preference, and patient choice.

To date, no predictive classifiers have been validated to select specific treatment regimens for individual patients with head and neck cancer in the curative setting. Crucially, while CRT may be viewed as equivalent to surgery and adjuvant treatment, the delivery of surgery and CRT in the subgroup of patients who receive triple therapy is viewed as escalation of treatment. Triple therapy results in more treatment complications and a higher quality of life detriment (10). Therefore, a biomarker would be particularly beneficial to select the patients, for example, those at higher risk, who could stand to benefit from this escalation of treatment, leading to an improvement in survival outcomes, whilst avoiding toxicity of unnecessary additional treatment in patients who do not benefit from escalation.

A systematic review and meta-analysis demonstrated that several biomarkers encode prognostic information for OPSCC (11). However, the included studies were found to be generally underpowered, with uncertainties around validation and reproducibility, differing scoring methods, and lack of consensus over “cut-off” points.

Recent research has developed prognostic classifiers based on p16 and HPV status, clinical stage (T and N categories), smoking history, alcohol consumption, and comorbidities (7, 8, 12, 13). These risk stratification approaches are currently recommended and used in routine clinical practice for diagnosis and prognosis (9, 14) and to stratify and recruit patients into clinical trials. However, they do not demonstrate predictive ability to select specific treatments.

Predictive biomarkers have recently been described for immunotherapy in the palliative treatment of patients with recurrent and/or metastatic head and neck cancer (15, 16). No predictive biomarkers for treatment in the curative setting are yet in routine clinical practice.

The aim of this study was to develop and validate a prognostic and predictive classifier for patients with OPSCC to guide treatment escalation with the combination of surgery and CRT. We also aimed to develop a classifier based exclusively on morphologic assessment and molecular analysis of standard formalin-fixed paraffin-embedded (FFPE) tissue samples, thereby eliminating the need for estimation of changing clinical TNM staging systems, self-reported lifetime tobacco consumption, and comorbidities for risk assessment. Our study was designed and reported according to the REMARK and TRIPOD guidelines (17, 18).

## Materials and Methods

We collated two retrospective longitudinal cohorts of consecutive patients 18 years of age or more, who had OPSCC treated with curative intent between January 1, 1999 and December 31, 2012, with either platinum-based CRT or surgery followed by adjuvant radiotherapy/chemoradiotherapy. For the development cohort, we collated 600 cases from ten secondary care head and neck cancer treatment centers in the United Kingdom and Poland. To undertake external (Grade 3) validation (18) of the biomarker classifier, we used an independent cohort (*n* = 385) of consecutive OPSCC patients undergoing curative treatment between 2002 and 2011, collated as part of a separate cohort, from three different centers from the previously published HPV UK Prevalence study (see Supplementary Methods for details; ref. 19). Baseline characteristics, treatment, outcome data, and formalin-fixed paraffin-embedded tumor samples were collated from patients’ medical records by clinicians who were blinded to biomarker analyses. The study received ethical approval from the National Research Ethics Service Committee West Midlands (10/H1210/9). The study was conducted in accordance with the Declaration of Helsinki.

### Laboratory methods

We selected our target biomarkers because they were shown in a systematic review to have evidence of prognostic ability specifically in OPSCC (11). We constructed tissue microarrays (1 mm diameter cores, up to 3 per case) using an automated machine (TMA Grand Master 3DHISTECH, Hungary) according to published guidelines (20). We used freshly cut 4-μm sections to perform haematoxylin and eosin (H&E) staining, immunohistochemistry (BCL-2, COX-2, Cyclin D1, EGFR external, HIF-1α, p16, PLK1, Survivin), and high-risk HPV DNA *in situ* hybridization (HR-HPV ISH). For each immunohistochemical test and the HR-HPV ISH, we undertook a validation process to assess if the TMA core staining was similar to corresponding whole section staining. To achieve this, we stained and scored whole sections from 10 tumors for each test. We then compared the scores from the whole sections with the mean score from the corresponding TMA cores. Tumor-infiltrating lymphocytes (TIL) were scored on H&E-stained whole sections as previously described (21). For further details, see Supplementary Materials and Supplementary Table S1.

### Scoring of biomarkers

After calibration and certification, at least three pathologists scored each biomarker independently, and were blinded to other test results and patient data. Scoring criteria are detailed in Supplementary Methods and Supplementary Fig. S1.

### Statistical analysis

#### Sample size and missing data

At an alpha = 0.001 and 80% power of detecting a hazard ratio of at least 1.6, and an interaction effect (i.e., ratio of the treatment hazard ratios of the two marker-defined groups) of at least 2.5, a sample size of up to 692 would be needed, depending on biomarker prevalence in each group. We first undertook a complete case analysis, with no imputation of missing data, then undertook imputation of missing values using four different methods using predictive mean matching (see Supplementary Methods for details).

#### Interobserver pathology estimates

We reconciled pathology estimates for molecular biomarkers by taking the mean score. For HR-HPV ISH, majority calls (0 or 1) were assigned. In case of a tie for HR-HPV ISH, we assigned a call of 1 to that patient. We evaluated interobserver concordance in a pair-wise manner (two observers at a time) using the Spearman correlation coefficient for continuous variables and Chi-square test for HR-HPV ISH.

#### Outcomes

The primary outcome measure was overall survival (OS), calculated as the time from diagnosis until death or censored at last clinical contact. The secondary outcome measure was disease-specific survival (DSS), calculated as the time from diagnosis until death due to locoregional persistence or recurrence or distant metastasis. Survival time was truncated at 5 years, and all subsequent survival analyses were performed on 5-year survival. The ties resulting (survival time = 5-year, event status = censored) from truncation were approximately 27% in both training and validation sets. These were handled using the Efron approximation (22) as implemented in the R survival package's function coxph().

We performed univariable and multivariable survival analyses (OS and DSS) using a Cox proportional hazards model. BCL2, COX2, Cyclin D1, EGFR-external, HIF1α, PLK1, and Survivin were treated as continuous variables. p16, HR-HPV DNA, TILs, age (<50 years, ≥50 years), gender, T-category, N-category, smoking status, surgery, radiotherapy, and chemotherapy were treated as factors/categorical. The predictive risk score of the survival models were grouped into either low- and high-risk groups (using training set's median risk score), or low-, intermediate-, and high-risk groups (using training set's risk score tertiles). The risk groups were tested for association with patient outcome (OS, DSS) using Cox proportional hazards model. Hazard ratios estimated from the Cox model were interpreted as an incremental increase in the hazard (event: death) in the intermediate- and high-risk groups relative to low-risk group.

To assess the differences in continuous variables between training and validation cohorts, mean and standard deviation were reported, and Wilcoxon rank-sum test was used for comparison. For categorical (factor) variables, counts were reported, and Fisher's exact test was used to assess the difference between training and validation cohorts. For further details, please see Supplementary Materials.

#### Model building, predictor handling, and risk groups

Using the preselected training cohort, we performed backward elimination on multivariable models with variable selection guided by Akaike Information Criterion (AIC) with adjustment for clinical covariates. The resulting refined models/predictors containing an optimal set of variables were then applied to the independent external validation cohort to predict individual (continuous) per-patient risk scores. The proportional hazards assumption for each predictor variable in the multivariable models created with step-wise variable selection (backward elimination) was tested by evaluating the Schoenfeld residuals against transformed time, as implemented by the R survival package's function cox.zph. All models reported a global chi-square *P* > 0.1 and a chi-square *P* > 0.01 for all predictor variables involved in the model, and therefore we did not assess for the presence of time dependent coefficients.

We applied the aforementioned models firstly to molecular biomarkers only to develop a model based solely on molecular biomarkers, then to clinical factors only (using TNMv7 and TNMv8 separately), and finally with all predictors together to assess whether a combination of clinical and biomarker factors produced a better model. As described above, our preference was for a biomarker-only model, which would be selected as the model to develop and validate if it was found to be equivalent to or better than the clinical only and the combined models. Information on cut-off point determination is included in Supplementary Materials. *P* values were estimated through Wald-test, log-rank test, or trend test as appropriate. *P* values for univariable models were adjusted for multiple comparisons using Benjamini–Hochberg procedure, thereby controlling for false discovery rates. The presence of pairwise interactions between biomarkers in the multivariable models was examined. For survival modelling, differences between the survival groups were assessed using the log-rank test. We undertook adjustment of the validation cohort results by year of diagnosis. We also adjusted the validation model with propensity scores to adjust for potential confounders arising from unbalanced distribution of covariates. To this end, we matched covariates (T-stage, N-stage, smoking status, and age at diagnosis) between the treatment groups (Surgery vs. CRT) using the R package MatchIt with parameters: method = “full”, distance = “glm”. The resulting weights were parameterised to the Cox model to estimate marginal HRs which are reported below. All data were analysed in R-statistical environment v3.2.4 using R packages: survival v2.38–3, survcomp v1.20.0, and MASS v7.3–45.

We also evaluated model performance. Discrimination was assessed by the Concordance Index (Harrell's C-Index). C-index of 0.5 represents random agreement, and 1.0 perfect agreement between model prediction and reality. Additionally, performance was also evaluated using sensitivity, positive predictive value (PPV, precision), and negative predictive value (NPV). Calibration plots were created for the training and validation sets (detailed in Supplementary Methods). Hazard regression (HARE) from R package polspline (v1.1.12) was used for the estimation of survival probabilities. Calibration analyses were performed using R package rms (v4.5–0). For further details, please see Supplementary Materials.

### Data Availability

The raw data are available upon request from the corresponding author and will require a data sharing agreement and ethics approvals for release of pseudo-anonymized data.

## Results

### Participants and baseline characteristics

We included data on a total of 985 subjects (Table 1; Supplementary Fig. S1). A total of 624 subjects were male and 225 were female, with a median age of 57 years (range: 19–91 years). A total of 501 subjects had T1-T2 disease (TNM-7), and 373 had N2b-N3 (TNM-7) nodal disease. Regarding treatment, 439 subjects had surgery: 41 received surgery alone, 240 received surgery and adjuvant radiotherapy, and 144 received surgery and adjuvant CRT. A total of 330 subjects received primary radiotherapy and platinum chemotherapy (Supplementary Table S2). No patient received cetuximab. The median OS of the cohort was 6.87 years [95% confidence interval (CI) = 6.41–9.03] and median follow-up was 5.03 years (95% CI = 4.73–5.21). The 5-year OS was 62.2% and the 5-year DSS was 73.8%. The distribution of missing data is shown in Supplementary Fig. S2 and Supplementary Table S3.

**Table 1.** Characteristics of the training and validation cohorts.

	Development set number (%)	Validation set number (%)	*P*_adj_
	600 (60.9%)	385 (39.0%)	
**Mean age, years (SD)**	57.5 (10.5)	58.2 (10.5)	0.719
**Gender**			0.876
Male	383 (63.8%)	241 (62.5%)	
Female	140 (23.3%)	85 (22.0%)	
N/A	77 (12.8%)	59 (15.3%)	
**Tumor category**			0.876
T1	118 (19.7%)	59 (15.3%)	
T2	204 (34%)	120 (31.2%)	
T3	120 (20%)	66 (17.1%)	
T4	129 (21.5%)	70 (18.2%)	
N/A	29 (4.8%)	70 (18.2%)	
**Nodal category**			0.008
N0	135 (22.5%)	92 (23.9%)	
N1	99 (16.5%)	39 (10.1%)	
N2	40 (6.7%)	8 (2.1%)	
N2A	54 (9%)	45 (11.7%)	
N2B	171 (28.5%)	90 (23.4%)	
N2C	49 (8.2%)	18 (4.7%)	
N3	28 (4.7%)	17 (4.4%)	
N/A	24 (4.0%)	76 (19.7%)	
**Smoking history**			0.341
Current	200 (33.3%)	137 (35.5%)	
Past	163 (27.1%)	92 (23.8%)	
Never	142 (23.6%)	69 (17.9%)	
N/A	95 (15.8%)	87 (22.5%)	
**Treatment**			
Radiotherapy	548	280	8.74×10^−4^
Chemotherapy	358	133	9.21×10^−12^
Surgery	264	175	0.022
**Recurrence**			0.493
Censored	352 (58.7%)	237 (61.5%)	
Events	142 (23.7%)	79 (20.5%)	
N/A	106 (17.6%)	69 (18%)	
**Overall survival**			0.002
Censored	373 (62.2%)	168 (43.6%)	
Events	193 (32.2%)	144 (37.4%)	
N/A	34 (5.6%)	73 (19%)	
**Disease-specific survival**			1.01×10^−4^
Censored	443 (73.8%)	201 (52.2%)	
Events	108 (18%)	101 (26.2%)	
N/A	49 (8.2%)	83 (21.6%)	
**Biomarkers, H-score (SD)**			
BCL2	64.5 (95.2)	60.0 (88.5)	0.639
COX2	146.6 (66.7)	148.1 (63.1)	0.876
CyclinD1	124.8 (107.0)	129.2 (102.2)	0.639
EGFR external	129.4 (79.3)	150.1 (81.9)	0.001
HIF1alpha	28.1 (46.6)	27.7 (44.4)	0.549
p16	163.9 (126.1)	151.1 (130.8)	0.596
PLK1	34.2 (30.8)	37.6 (29.5)	0.041
Survivin	62.9 (37.6)	71.9 (36.9)	0.002
**HR-HPV ISH**			0.83
0	368 (61.3%)	245 (63.6%)	
1	160 (26.7%)	113 (29.4%)	
N/A	72 (12.0%)	27 (7.0%)	
**TILs**			0.639
1	89 (14.8%)	67 (17.4%)	
2	192 (32.0%)	177 (45.9%)	
3	119 (19.8%)	94 (24.4%)	
N/A	200 (33.3%)	47 (12.2%)	

Note: Numbers in parentheses are percentages where specified, otherwise standard deviation. All staging was performed according to AJCC/UICC TNM 7^th^ edition manual.

### Distribution of biomarker scores and interobserver correlation between pathologists

**Figure 1.**
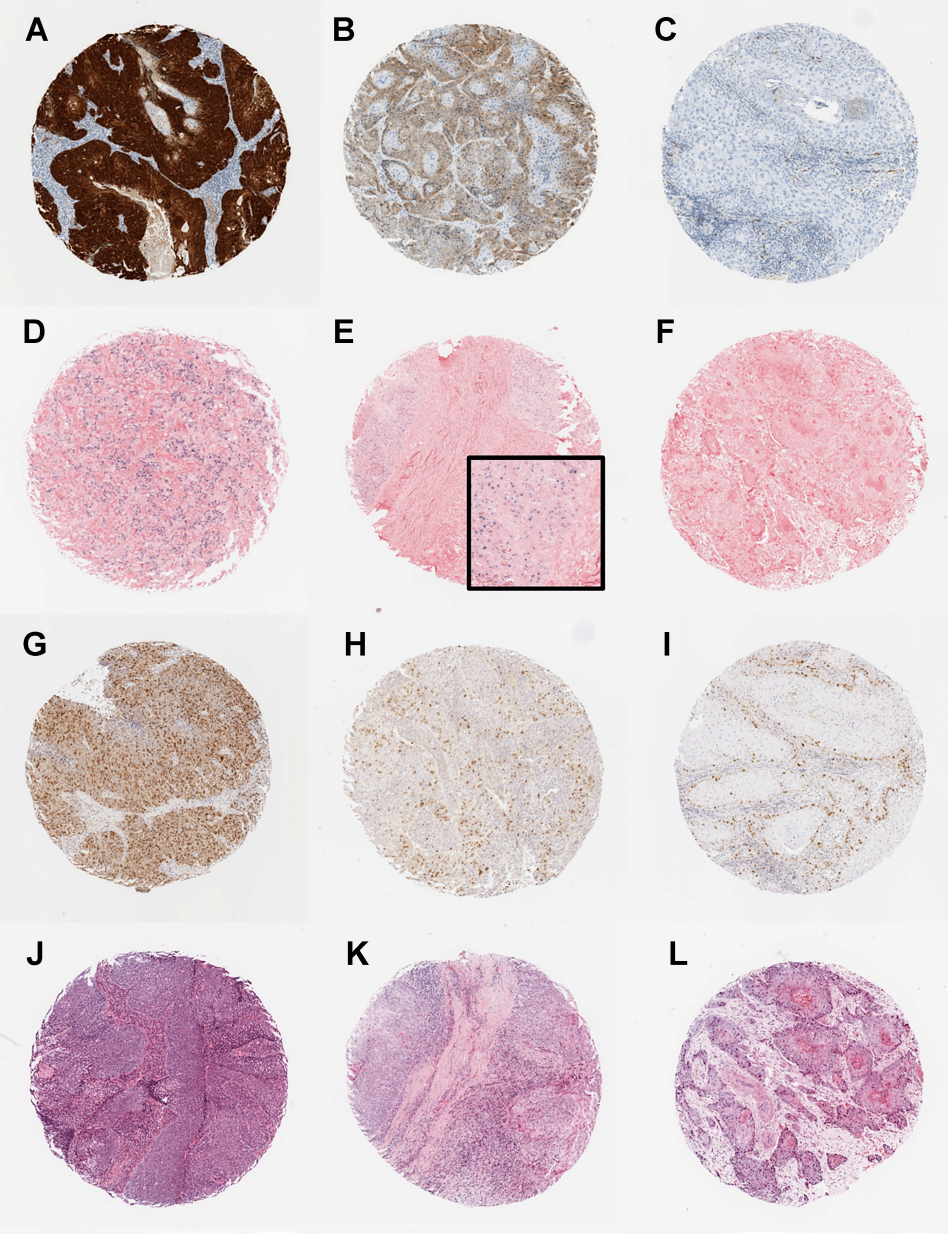
Photomicrographs showing examples of the biomarkers in the predictive classifier: p16 immunohistochemistry (**A–C**), high-risk HPV *in situ* hybridization (**D–F**), survivin immunohistochemistry (**G–I**), and TILs (**J–L**). **A,** p16-positive tumor showing strong and diffuse nuclear and cytoplasmic staining. **B,** p16-negative tumor showing weak and diffuse cytoplasmic staining. **C,** p16-negative tumor with no staining. **D–E,** High-risk HPV-positive tumors showing diffuse nuclear and cytoplasmic staining (**D**) and punctate nuclear staining (**E**). **F,** High-risk HPV-negative tumor with no staining. **G–I,** Survivin staining showing tumors with high (**G**), medium (**H**), and low (**I**) H-scores. **J–L,** Cases with high (**J**), moderate (**K**), and low (**L**) TILs.

For each biomarker (Fig. 1; Supplementary Table S1), we measured interobserver agreement between three or four pathologists. Interobserver correlation between scorers was generally high; mean Spearman *ρ* (rho) > 0.75 (range: 0.65–0.97, *P* < 10^−3^), with the exception of COX-2, which showed lower agreement Spearman *ρ* (rho) of 0.58–0.72 (Supplementary Table S1). The distribution of H-scores for each biomarker are given in Supplementary Fig. S3 and Supplementary Table S4.

### Univariable assessment of molecular biomarkers and clinical factors

**Figure 2.**
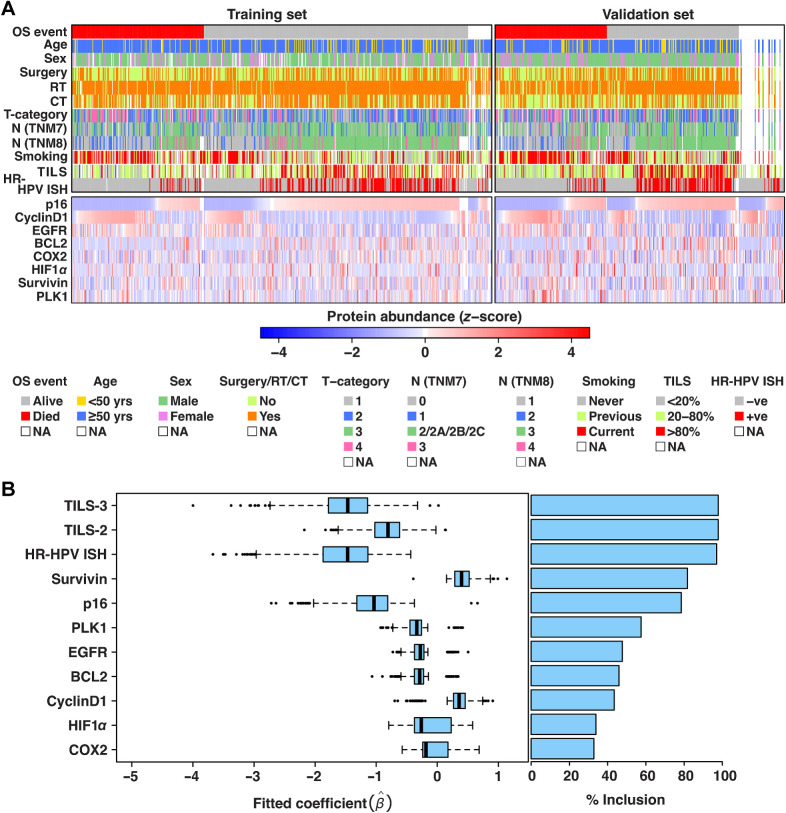
**A,** Heatmaps summarizing the molecular and clinical data from training and validation sets. Rows contain clinical covariates and molecular biomarkers, while columns contain patients. Protein abundance H-scores (range: 0–300) were divided by 30 and *Z* transformed (*μ* = 0, *σ* = 1) for training and validation sets separately. Column order in each of the training and validation sets was determined by first sorting (low to high *Z*-score) on the protein biomarkers one by one (bottom up) and then OS event. **B,** Box plots displaying range of fitted coefficients including 25^th^ percentile (Q1), median, and 75^th^ percentile (Q3). To test the stability of the variables selected in the multivariable model, bootstrapping was performed on the training cohort (1,000 times) and Cox proportional hazards model was fitted with backward elimination on each subset. For each bootstrap iteration, coefficients of the resulting variables selected by the model are displayed in the box plots alongside their % frequency of inclusion over 1,000 iterations. Bootstrapping results confirmed the relative importance of variables in our original model (Supplementary Table S6), as these were ranked among the top recurrently selected variables over 1,000 iterations. Upper whisker of the box plots indicates: min (max(x), Q3 + 1.5 × IQR) and lower whisker indicates: max (min(x), Q1–1.5 × IQR) where IQR = Q3−Q1. Color key: TILS-3 = high, TILS-2 = moderate, TILS-1 = low.

For the purpose of developing risk predictors for OPSCC and subsequent independent validation, we used two separate training and validation datasets. Molecular and clinical characteristics between the training and validation cohorts were similar (Fig. 2; Table 1). There were differences in the treatment proportions received in each set. In the validation set, more cases received surgery (54.5% vs. 45.1%, *P* = 0.02) and fewer received chemotherapy (41.96% vs. 67.6%; *P* < 0.001) or radiotherapy (86.96% vs. 94.5%; *P* < 0.001; expressed as percentage of known treatment type).

Univariable survival analysis in the pre-assigned training set showed statistically significant associations between OS (the primary outcome) and the following factors: T category, N category, smoking status, age, radiotherapy, surgery, HR-HPV ISH, p16, BCL-2, Cyclin D1, EGFR external, and TILs (adjusted *P* < 0.05; Supplementary Table S5). Of the molecular biomarkers, HR-HPV ISH, p16, TILs, and BCL-2 were associated with good outcome while Cyclin D1 and EGFR external were associated with poor outcome. As expected, smoking was a prognostic marker of poor outcome as well as T category. Amongst the treatment variables, both radiotherapy and surgery were associated with increased survival.

### Development and validation of a multivariable prognostic model for OPSCC based on molecular biomarkers

**Figure 3.**
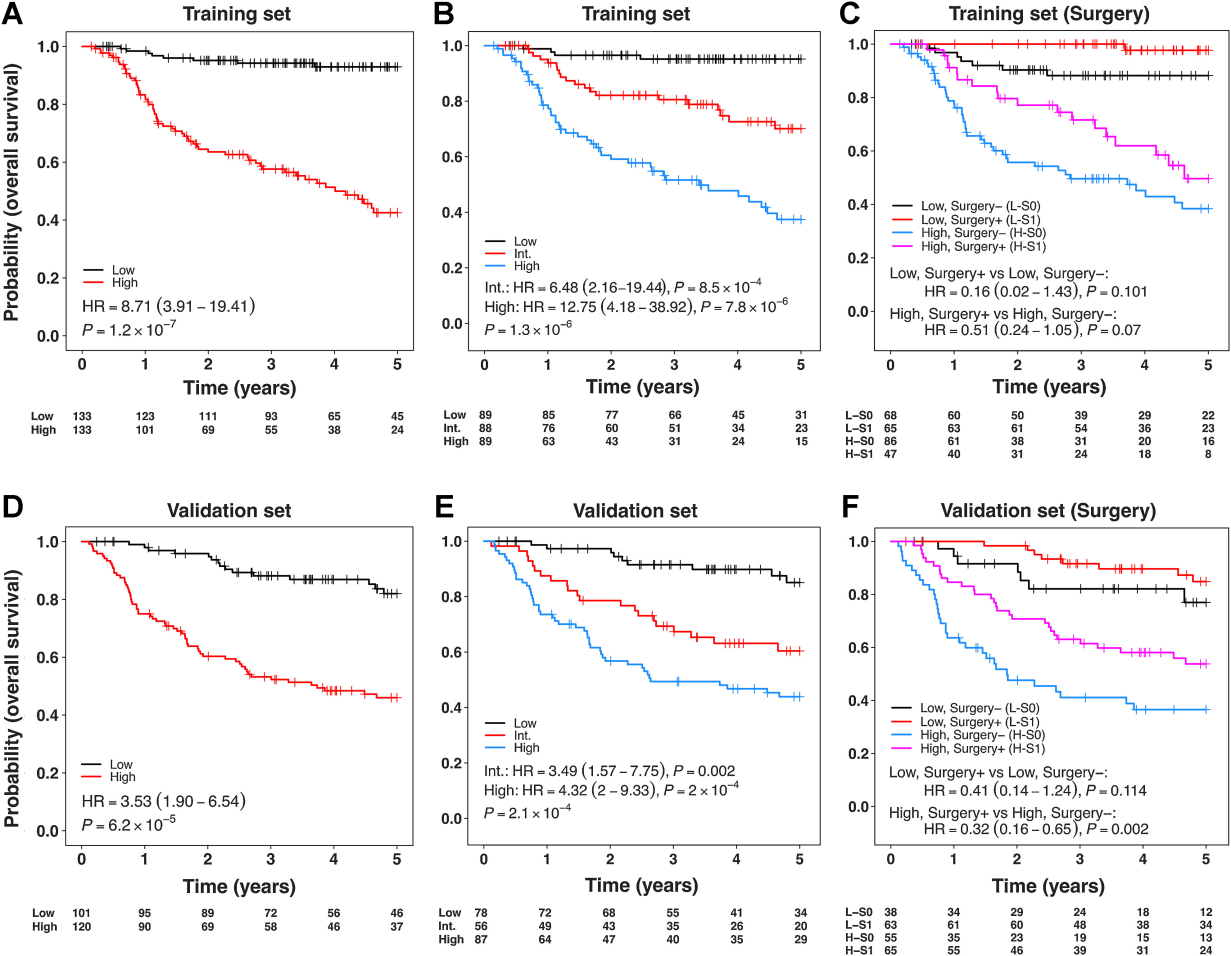
**A–C,** Prognostic and predictive assessment of risk groups predicted by multivariable survival model (trained with backward elimination using AIC) based on molecular biomarkers only with OS, when applied to the training cohort. **D–F,** Prognostic and predictive assessment of risk groups predicted by the molecular biomarkers only multivariable model, when applied to the validation cohort. Risk groups in the validation cohort were created using the thresholds (two-group classification: median risk score; three-group classification: tertiles of risk score) derived from the training set. Color key **A, D:** black = low-risk group, red = high-risk group; **B, E:** black = low-risk, red = intermediate (Int.)-risk, Blue=high-risk group; **C, F:** Red = low-risk surgery, black = low-risk no surgery, pink = high-risk surgery, blue = high-risk no surgery group. Models were adjusted for clinical covariates: T-category, N-category, smoking status, age, radiotherapy, and chemotherapy.

To rationalize clinical heterogeneity in OPSCC, we created a multivariable survival model (with OS) based on molecular biomarkers using the training cohort. Following optimal selection of molecular markers (see Materials and Methods), the final model was composed of p16 and survivin IHC, HR-HPV DNA *in situ* hybridization (ISH), and TILs (Supplementary Table S6). The coefficients for p16, HPV, and TILs were associated with good outcome, while survivin showed association with poor outcome, demonstrating parameter inference and directionality of effects in line with findings from previous studies. The robustness and directionality of effect size of these molecular biomarkers were further tested by creating an additional 1,000 multivariable models using bootstrapping on the training cohort, which confirmed p16, survivin, HR-HPV ISH, and TILs as the top four recurrently selected features (inclusion > 60% of the models; Fig. 2B). There were no significant pairwise interactions between any of the four selected biomarkers. The model was prognostic in the training set when predicted risk scores were dichotomized and trichotomized into unbiased equal-sized risk groups (Fig. 3A and B; see Materials and Methods). The model showed potential of improved survival following surgical treatment, albeit nonsignificant, for both high-risk patients (HR = 0.51; 95% CI = 0.24–1.05; *P* = 0.07) and low-risk patients (HR = 0.16; 95% CI = 0.02–1.43; *P* = 0.101; Fig. 3C).

When tested in the validation cohort, the model was prognostic for OS following adjustment for clinical covariates (T-category, N-category, smoking status, age, radiotherapy, chemotherapy). Two-group HR was 3.53, 95% CI = 1.90–6.54, *P* = 6.2 × 10^−5^ and three-group was *P* = 2.1 × 10^−4^ (Fig. 3D and **E**). In the two-group classification, 54.2% of cases were classified as high-risk. Acknowledging the 13-year window (1999–2012) across which patients involved in this study were diagnosed, we tested the possibility of confounding impact of changes in treatment strategies over this period. To this end, we further adjusted the model for the year of diagnosis when evaluating the prognostic association of risk groups in the validation cohort. Both two- and three-group classifications remained independent predictors of patient outcome (OS; two-group HR: 3.52; 95% CI = 1.90–6.55; *P* = 6.7 × 10^−5^ and three-group: *P* = 2.3 × 10^−4^).

In the validation cohort, the model was predictive of improved survival following treatment with surgery and chemotherapy over CRT alone for high-risk patients (HR = 0.32, 95% CI = 0.16–0.65, *P* = 0.002; Fig. 3F), but was not statistically significant in the low-risk patients (HR = 0.41, 95% CI = 0.14–1.24, *P* = 0.114; Fig. 3F). The 3-year OS for the low-risk patients receiving surgery was 91.6% and for those not receiving surgery was 82.1%. For the high-risk patients, the 3-year OS was 63.1% for those receiving surgery and 41.1% for those who did not (Fig. 3F; Table 2). When further adjusted for year of diagnosis, the results remained highly similar (high-risk surgery + adjuvant treatment versus high-risk CRT: HR: 0.32, 95% CI = 0.16–0.65, *P* = 0.002 and low-risk surgery + adjuvant treatment versus low-risk CRT: HR: 0.41, 95% CI = 0.14–1.23, *P* = 0.113). Data on heterogeneity of risk factors across the high- and low-risk groups did not show any confounders (FDR-adjusted *P* ≥ 0.05) except for T-category where the T2 stage was associated with surgery in the high-risk patients (FDR-adjusted *P* < 0.05; Supplementary Table S7; Supplementary Fig. S4C–S4F). When we adjusted the model for propensity scores of the covariates T-stage, N-stage, smoking status, and age at diagnosis, the results further highlighted the benefit of surgery in the high-risk group (low-risk surgery + adjuvant treatment versus low-risk CRT: HR = 0.5, 95% CI = 0.1–2.38, *P* = 0.384, and high-risk surgery + adjuvant treatment versus high-risk CRT: HR = 0.34, 95% CI = 0.17–0.67, *P* = 0.002).

**Table 2.** Clinical examples of the OS model.

	All		Surgery ± Radiotherapy		Chemoradiotherapy	
	3-year	5-year	3-year	5-year	3-year	5-year
Biomarker panel	OS (%)	OS (%)	OS (%)	OS (%)	OS (%)	OS (%)
HPV+, p16+, survivin-low, TILs-high	96.77	96.77	100.00	100.00	100.0	100.00
HPV+, p16+, survivin-high, TILs-high	89.93	89.93	95.65	95.65	90.40	90.40
HPV+, p16+, survivin-low, TILs-low	86.23	78.84	91.48	81.25	90.00	82.50
HPV+, p16+, survivin-high, TILs-low	84.79	77.35	94.03	79.00	88.54	85.26
HPV−, p16−, survivin-low, TILs-high	40.00	30.00	50.00	33.33	20.00	20.00
HPV−, p16−, survivin-high, TILs-high	66.67	42.86	85.71	68.57	57.14	NA
HPV−, p16−, survivin-low, TILs-low	49.35	36.93	58.27	40.06	51.29	43.14
HPV−, p16−, survivin-high, TILs-low	41.91	34.68	50.18	50.18	36.44	32.80

Note: This table demonstrates the 3- and 5-year OS for the subjects with different combinations of biomarker signatures, having received surgery±radiotherapy or radical chemoradiotherapy.

The model showed a Concordance index (C-index) of 0.73 (95% CI = 0.68–0.78), with sensitivity = 0.80 (95% CI = 0.71–0.90) and PPV = 0.63 in the validation set and was reasonably calibrated for both training and validation sets (Supplementary Fig. S4A and S4B; Supplementary Methods).

When tested with DSS as an outcome in the validation set, the model was again prognostic (HR = 3.17, 95% CI = 1.56–6.46, *P* = 0.001; Supplementary Fig. S5A), independent of clinical covariates (T-category, N-category, smoking status, age, radiotherapy, chemotherapy). The model was also predictive of benefit from surgery and adjuvant therapy over CRT alone for the high-risk group (HR = 0.27, 95% CI = 0.12–0.62, *P* = 0.002), and showed a similar trend for the low-risk group (HR = 0.33, 95% CI = 0.10–1.14, *P* = 0.07; Supplementary Fig. S5B).

### Alternative survival models based on clinical variables

**Figure 4.**
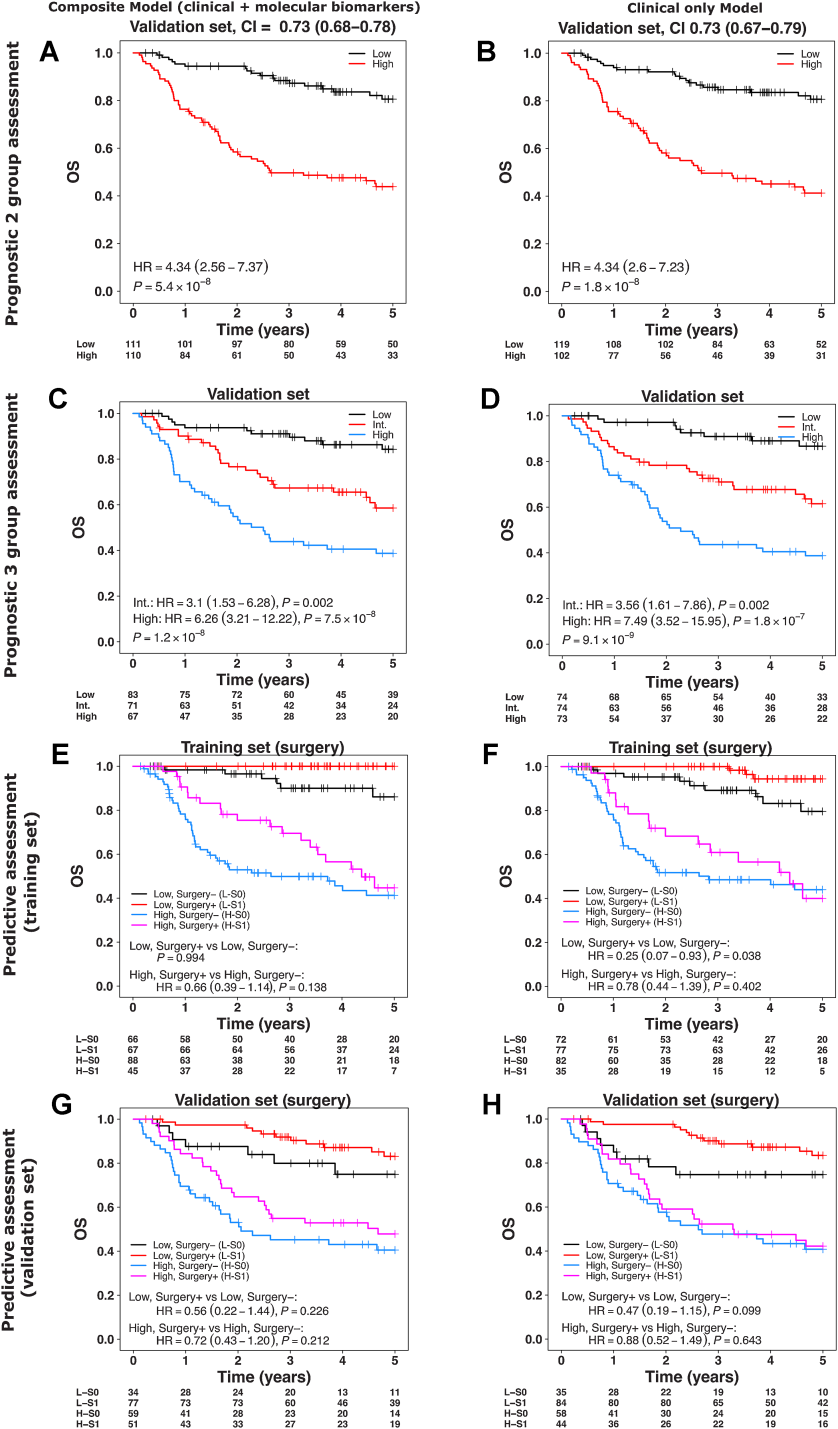
Multivariable survival modelling (trained with backward elimination using AIC) for composite (molecular biomarkers + clinical factors) and clinical factors only with OS. **A–D,** Assessment of composite and clinical only models in the validation cohort split into two and three risk groups. **E–H,** Assessment of composite and clinical only models’ predicted risk groups (low- and high-risk) stratified by surgery in the training (**E** and **F**) and validation cohorts (**G** and **H**). Risk groups in the validation cohort were created using the thresholds (two-group classification: median; three-group classification: tertiles) derived from the training set. In **E**, the estimate of HR (95% CI) was not possible due to absence of events in low, surgery+ group. Color key: same as Fig. 3.

To investigate the prognostic ability of clinical variables by their inclusion alongside molecular biomarkers in a multivariable model and on their own, we created two alternative models using the training cohort: (i) combined clinical and molecular biomarkers model and (ii) clinical only model. Following feature selection in the training cohort using backward elimination, the refined version of the combined clinical and molecular biomarkers model contained all variables except COX2, EGFR external, p16, and age (Supplementary Table S8). The clinical only model excluded age during the training stage (Supplementary Table S9). Both models were prognostic for OS and DSS in the validation set (Fig. 4A–D; Supplementary Fig. S5C and S5D). In comparison with the molecular biomarkers model, neither the clinical only nor the combined (clinical and molecular biomarkers) models were predictive of benefit from combined treatment in the high-risk group for OS (Fig. 4E–H). However, the clinical only model was predictive of benefit from surgery with adjuvant therapy in the low-risk group for DSS (HR = 0.32, 95% CI = 0.11–0.9, *P* = 0.031; Supplementary Fig. S5E and S5F). We created an additional clinical only model by substituting TNMv8 with TNMv7 (Supplementary Table S10), which was also prognostic, but not predictive of surgical benefit in the high-risk group for OS or DSS (Supplementary Fig. S6). Overall, the clinical only model as well as the combined model did show a trend of predictive associations with treatment, albeit less pronounced, yet similar in direction as identified by the molecular biomarkers model.

To assess the impact of missing values in the molecular biomarkers (Supplementary Fig. S2), we performed imputation using predictive mean matching in four different ways. Using the imputed datasets, both the multivariable models of molecular biomarkers only and the composite markers were retrained. When tested in the validation cohort, both sets of models (with and without imputation) demonstrated very similar C-index, PPVs, and NPVs suggesting robustness of our complete cases models (Supplementary Table S11).

## Discussion

Several prognostic classifiers have been developed for OPSCC, but none are predictive for treatment selection in the curative setting. Our PREDICTR-OPC classifier was not only prognostic, but also shows some utility in the selection of treatment. Those patients categorized as high-risk by the classifier demonstrated significantly better OS if they received treatment escalation by surgery with CRT, compared with CRT alone, indicating that these high-risk patients may benefit from treatment escalation with triple therapy. Although patients classified as low-risk also showed a trend of potential benefit with triple therapy, this was not statistically significant. This is likely due to lack of statistical power, which impacts both low- and high-risk groups, especially when they are split into subgroups stratified by surgery. Further validation in an adequately powered study is required. Importantly however, triple therapy results in significantly worse functional outcomes (10); therefore, a large effect size is needed to justify its use. Even if there was a statistically significant difference in survival between the two treatments in the low-risk patients, the absolute difference in OS is only 9%, which arguably may not justify the additional toxicity and functional detriment of triple therapy in this low-risk group of patients. On the other hand, the absolute difference in OS between the two treatment regimens in the high-risk arm was 20%. This is a very large improvement in survival—more than any new treatment in head and neck cancer in the last 30 years—and so would justify the use of triple therapy and its attendant additional morbidity. The development of a prognostic-predictive biomarker classifier provides the opportunity to guide selection based on biology and treatment efficacy, rather than simply on clinician or patient preference. This holds the potential for an improvement in survival outcomes in the longer term, which is especially pertinent for the high-risk group.

The main limitation of other classifiers has been that they were only prognostic. Having been developed on small cohorts with no validation, many also suffered from significant methodologic weaknesses (11). In addition, some depended on clinical factors and the estimation of lifetime tobacco consumption (7, 8), which may be prone to underreporting (23) and variability, leading to inaccurate risk assessment. To address these limitations, we extensively tested our molecular biomarker for treatment associations, an approach that demonstrated higher predictive power over the alternative clinical only and the combined clinical and biomarker classifiers developed in our study.

We set out to develop a classifier based exclusively on biomarkers to avoid clinical factors, whose estimation and reporting can be variable. This may partly explain why the predictive models that incorporated clinical factors showed higher relative benefit from triple therapy in lower-risk groups than the higher-risk groups, which is counterintuitive and not clinically usable. The biomarker-only model on the other hand demonstrated relative and absolute benefits that are broadly intuitive, in line with current knowledge and expectations, and which would be clinically usable.

The PREDICTR-OPC classifier is underpinned by biologically relevant parameters. p16 immunohistochemistry and high-risk HPV-DNA *in situ* hybridization are indicators of HPV-driven oncogenesis of OPSCC (7, 8). HPV-driven tumors appear to be more responsive to treatment, possibly due to a lower incidence (24) or different pattern (25) of mutations. While p16 is a surrogate for HPV infection (24), recent data suggest that there is added utility in assessing both p16 and HPV status of the tumor, because a proportion of p16-positive tumors may be HPV-DNA negative, and appear to behave more like the higher-risk p16-negative group of patients (26, 27). TILs is widely accepted as a surrogate indicator for the immune response to the cancer, as tumor-infiltrating T-lymphocytes are the effector cells of this immune response (28). TILs has been shown by Ward and colleagues (21) and others (29) to be an independent and reproducible prognostic marker for OPSCC. Survivin is a member of the inhibitor of apoptosis (IAP) gene family, which encode regulatory proteins that prevent apoptosis. It is generally deregulated in cancer (30) and is known to be over-expressed in OPSCC (11, 31). A meta-analysis of survivin expression in oral cancer demonstrated that increased expression was associated with poor prognosis (HR death 1.62; ref. 31). As a downstream marker of the inhibition of apoptosis, survivin may indicate more tumor kill when the tumor is treated by cisplatin which induces apoptosis through several mechanisms. This may explain some of the added benefit seen from the use of cisplatin in the high-risk group of patients who would likely have overexpression of survivin. It should be noted however, that while survivin was a statistically significant predictor of survival in the multivariable analysis, its contribution to the predictive classifier is very small. This may explain why it did not show significant association with outcomes in the univariable analysis.

Combining these biological factors, therefore, may provide a relatively comprehensive evaluation of the hallmarks of that particular tumor, encompassing molecular pathogenesis and mutation status (HPV, p16), immune response to the tumor (TILs), and the state of cell-cycle control and apoptotic mechanisms (survivin, p16). As our molecular biomarker signature outperforms the new clinical models that incorporate p16 expression (TNMv8 classification) in improved sensitivity and ability to predict benefit from surgery for OS, it also appears to be providing additional information over and above assessment of this parameter.

High-risk patients treated with a combination of surgery and adjuvant chemo/radiotherapy therapy demonstrated improved OS and DSS. One potential explanation is that they benefit from treatment escalation with triple therapy. Another explanation could be that patients who receive surgery may have lower-stage disease, or better performance status, resulting in better outcomes. To examine the effect of tumor size, we analyzed the distribution of important potential factors that may affect survival outcome across the low- and high-risk groups, stratified by surgery. Gender, N (nodal) category, smoking status, and HPV status showed no significant differences between the groups. Only T category was significantly differently distributed (Supplementary Table S7), suggesting that patients with T1-T2 were more likely to be selected to receive surgery, as would be expected clinically. To account for any confounding due to tumor size (T category) on OS, we therefore examined the differences in survival between the surgical and nonsurgical groups in each of the T1–4 categories (Supplementary Fig. S4C–S4F). We found significant differences in survival in favor of combined surgery and CRT in the T2 category, and trends in T1 and T3 categories, which did not reach significance despite widely separated KM curves (likely due to low case numbers). No such difference due to combined treatment was identified in the high-risk patients with T4 disease, due to the higher incidence of distant metastases, which cannot be cured by local surgical intervention. This suggests a real effect and not simply a confounder. We did not collect performance status. However, the proportion of patients who were current or ex-smokers (a main determinant of comorbidity in this patient group) was very similar (84% versus 92%) between the two groups, suggesting that performance status would not be the main contributor.

Our study's main limitation is the retrospective collection of patient data (especially smoking status) and lack of randomization, necessitated by the lack of any prospective randomized studies. There is currently no other way to develop and validate such a classifier, as no studies have been able to successfully randomize between surgery and CRT in OPSCC. The potential for bias in a retrospective design of the study was mitigated by adherence to best practice in biomarker studies and the TRIPOD guidelines (18), the use of a “hard” endpoint of death which is reliably ascertained and documented, and data collection by clinicians and scoring by pathologists who were blinded to biomarker results and patient outcomes, respectively.

Furthermore, although we observed a significant benefit from combined triple treatment in the predicted high-risk group, a similar but statistically nonsignificant trend was also observed in the low-risk group. This could be due to limited statistical power for this subgroup. However, clinical management of low-risk group patients, who have very good survival outcomes with dual modality treatment, would require weighing the toxicity and functional deficit of additional treatment and any survival benefit from triple therapy.

A particular strength of our study was that we undertook stage 3, external independent validation (18), demonstrating applicability and efficacy on a separate independent cohort which had different treatment characteristics to the development cohort. The prognostic and predictive analyses demonstrated the usual patterns of higher prognostic and predictive power in the training cohorts, where overfitting is expected, followed by reduced prognostic power in the external, independent validation cohort. Final stage validation in a prospective cohort or randomized trial, using full FFPE sections, is now necessary, and is currently underway, before the classifier can be adopted in routine clinical practice.

The laboratory costs of our PREDICTR-OPC classifier (around £100/$140 per patient) are low when considered in the context of the cost of the patient's entire head and neck cancer treatment pathway, reported to be up to £19,778 ($28,000) in the United Kingdom and between $5,000 and $35,000 in the USA. Furthermore, by personalizing therapy and maximizing treatment efficacy, the cost benefit of this classifier is likely to be high.

In conclusion, the development of the PREDICTR-OPC classifier provides the promise of personalizing therapy by escalating treatment for patients with higher-risk OPSCC, guided by biological parameters of treatment efficacy, rather than only on clinician preference or treatment availability. Validation in a prospective setting is now underway in preparation for clinical adoption.

## Supplementary Material

Supplementary Data 1 Combined Supplementary Methods, Figures and Tables
