# Supplementary material for: Developing and Validating a Multivariable Prognostic-Predictive Classifier for Treatment Escalation of Oropharyngeal Squamous Cell Carcinoma: The PREDICTR-OPC Study
Source: Clin Cancer Res. 2023 Oct 23;30(2):356–67. doi: 10.1158/1078-0432.CCR-23-1013 (PMC10792360; doi:10.1158/1078-0432.CCR-23-1013)
Supplement: Supplementary Data 1 — Combined Supplementary Methods, Figures and Tables [file ccr-23-1013_supplementary_data_1_suppds1.pdf]

Supplementary methods

Participants

For the development cohort, we collated 600 cases from ten secondary care head and neck cancer treatment centres in the UK and Poland. Bradford Teaching Hospitals NHS Foundation Trust (n=26), Heart of England NHS Foundation Trust, Birmingham (n=39), John Radcliffe Hospital, Oxford (n=103), The Christie NHS Foundation Trust, Manchester (n=65), The Greater Poland Cancer Centre, Poznan (n=43), The Royal Marsden NHS Foundation Trust, London (n=178), The Royal Wolverhampton NHS Trust (n=81), Queen Victoria Hospital NHS Foundation Trust, East Grinstead (n=15), University Hospital Birmingham NHS Foundation Trust (n=32), Walsall Healthcare NHS Trust, (n=18).

To undertake external (Grade 3) validation of the biomarker classifier, we used an independent cohort (n=385) of consecutive oropharyngeal cancer patients undergoing curative treatment between 2002 and 2011, collated as part of the HPV UK Prevalence study[1], from Aintree University Hospital NHS Foundation Trust, Liverpool (n=146), University Hospitals Bristol NHS Foundation Trust (n=66), and University Hospitals Coventry and Warwickshire NHS Trust (n=115). 58 samples in the validation cohort were missing data on centre of origin. The centres were selected to ensure a mix of geographic location, centre size and institutional treatment protocols.

Patients were all treated with curative intent, with either chemoradiotherapy, radiotherapy alone or surgery with or without radiotherapy/chemoradiotherapy. In accordance with UK National Multidisciplinary Guidelines[2] all patients underwent regular follow-up after treatment and were reviewed every 6-8 weeks in first year, every 8-12 weeks in second year, and every 4-6 monthly thereafter, for a period of at least 3 years or until death.

Baseline characteristics, treatment, and outcome data were collated from

patients' medical records by clinicians who were blinded to biomarker analyses. All staging was performed according to the AJCC/UICC TNM 7th edition clinical staging manual[3] and converted to the new staging system following the publication of AJCC/UICC TNM 8th edition[4]. Formalin-fixed paraffin-embedded tumour samples from diagnostic biopsies were obtained for each patient. The study received ethical approval from the National Research Ethics Service Committee West Midlands (10/H1210/9).

Laboratory methods

Haematoxylin and eosin (H&E) stained sections from formalin-fixed paraffin-embedded tissue blocks were reviewed by a pathologist to confirm the diagnosis of squamous cell carcinoma and TMAs were constructed using an automated TMA machine (TMA Grand Master 3DHISTECH, Hungary) according to published guidelines[5]. Up to three 1mm cores were obtained from each of the donor blocks and transferred to a recipient block. H&E-stained sections of the TMAs were examined to quality assure tumour sampling prior to downstream testing. Freshly cut 4µm sections were used to perform immunohistochemistry (BCL-2, COX-2, Cyclin D1, EGFR external, HIF-1α, p16, PLK1, Survivin) and high-risk HPV DNA *in situ* hybridization (HR-HPV ISH). The assays used were either verified diagnostic tests performed in a pathology laboratory with Clinical Pathology Accreditation (CPA (UK) Ltd) or 'research use only' reagents that were optimized for the study (**Supplementary Table S1**).

Scoring of biomarkers

All pathologists attended a training and calibration meeting and underwent certification by scoring three test TMAs before contributing data to the study. At least three pathologists scored each biomarker independently and were blinded to other test results and patient data. Immunohistochemistry was assessed by assigning an intensity score (0, no staining; 1, weak; 2, moderate; 3, strong) and the percentage (0-100% in 5% increments) of malignant cells staining at each intensity. These parameters were used to calculate an H-score [product of intensity and percentage; formula (0xa%)+(1xb%)+(2xc%)+(3xd%)=H-score (continuous variable 0-300)][6]. HR-HPV ISH was stained using Ventana INFORM

HPV III Family 16 probe (B), which detects HPV-16, -18, -31, -33, -35, -39, -45, -51, -52, -56, -58, -66, and was scored using a binary classification (positive vs. negative).

Tumour infiltrating lymphocytes (TILs) were scored on H&E-stained whole sections as previously described[7]. Briefly, on scanning magnification (x2.5 objective) one of the following categories were assigned: high TILs (diffuse; present in >80% of tumour/stroma), moderate TILs (patchy; present in 20-80% of tumour/stroma) or low TILs (weak/absent; present in <20% of tumour/stroma) (**Figure 1**).

Cut points for variables

Since there was no known clinical proportion of high-risk patients at the exploratory stage, we consulted with a group of head and neck clinicians regarding clinical utility, and two sets of risk groups were developed: One with two risk groups (low-risk and high-risk groups) achieved by dichotomising the risk scores of the training set, using the median risk score of the training cohort as cut-off for the validation cohort. The other model had three risk groups (low-risk, intermediate (Int.)-risk and high-risk groups), obtained by dividing the risk scores of the training cohort into tertiles; which were then used as cut-offs for the validation cohort.

H-score of continuous variables (BCL2, COX2, Cyclin D1, EGFR-external, HIF1α, PLK1, Survivin) was scaled to 0-10 by dividing H-score by 30, as previously described[8], and further transformed to z-scores (mean = 0, standard deviation = 1) to enable the input scores for this study to be comparable to scores arising from different clinical cohorts and distributions. p16 was dichotomised into positive and negative categories according to a previously described, clinically validated cut off ('strong and diffuse nuclear and cytoplasmic staining present in ≥70% of the tumour'; H-score equivalent ≥2 intensity x ≥70% = H score ≥140)[9,10].

Missing value imputation

We first undertook a complete case analysis, with no imputation of missing data, since the survival analysis demonstrated no statistically significant

differences in survival between subjects with complete data sets and those with missing data (**Supplementary Table S3, Supplementary Figure S2**). We then undertook imputation of missing values for molecular variables in four different ways using predictive mean matching and compared the models proposed by complete data sets with those created using imputation of missing values. Four imputation methods were based on combination of two parameters: 1) pmmtype = {1, 2} and 2) boot.method = {simple, approximate bayesian} using R package Hmisc v4.1-1. For details of parameters, see R function Hmisc::aregImpute. (**Supplementary Table S11**).

Outcomes

A Cox proportional hazards model was used for survival analyses. The outcome of the model was interpreted as a hazards ratio, which represents an incremental increase in the hazard (event: death of any causes (OS) or disease specific (DSS)) in the intermediate- and high-risk groups relative to low-risk group. Positive coefficients in the model indicate association with poor outcome, while negative coefficients indicate association with good outcome.

Model building, predictor handling and risk groups

All models were trained exclusively on a pre-assigned training cohort (60% of data) only. The independent validation cohort (40% of data) was used to predict individual patient risk scores using the models trained on the training cohorts. H-score scaling and z-transformation was performed on training and validation sets separately. For continuous variables, mean and standard deviation was estimated, and Wilcox rank-sum test was used to assess the difference between training and validation cohorts. For categorical (factor) variables, counts were reported, and Fisher's exact test was used to assess the difference between training and validation cohorts. P-values were adjusted for multiple comparisons using Benjamini-Hochberg procedure thereby controlling for false discovery rate. For survival modeling, differences between the survival groups were assessed by log-rank test.

Univariable models were created using a Cox proportional hazards model. BCL2, COX2, Cyclin D1, EGFR-external, HIF1α, PLK1 and Survivin were treated as continuous variables. p16, HR-HPV DNA, TILs, age (<50, >=50), gender, T-

category, N-category, smoking status, surgery, radiotherapy and chemotherapy were treated as factors using the lowest category as baseline group. We examined the aforementioned models with biomarkers only to develop a model based solely on biomarkers, then with all predictors, including clinical factors, to assess whether a combination of clinical and biomarker factors produced a better model.

Multivariable predictors were created using a Cox proportional hazards model with backward elimination performed on the training cohort, with variable selection guided by Akaike Information Criterion (AIC) using *k* (degrees of freedom) = qchisq(p = 0.25, 1, lower.tail = FALSE); see R manual for details. The final predictors with a reduced set of variables created exclusively on training cohorts were applied to the independent validation cohort to predict individual (continuous) per-patient risk scores. Positive coefficients in the model indicate association with poor outcome, while negative coefficients indicate association with good outcome.

We developed two sets of risk groups: one with two risk groups (low- and high-risk groups) by dichotomising the risk scores of the training set; and another with three risk groups (low-, intermediate- and high-risk groups) by dividing the risk scores into tertiles. For the low- and high-risk grouping, the median risk score of the training cohort was used as cut-off in the validation cohort. For low-, intermediate- and high- risk grouping, the tertiles of risk scores derived from the training cohort were used as cut-offs in the validation cohort.

The associations of the risk groups with the outcome (OS, DSS) were tested using a multivariable Cox proportional hazards model with adjustment for clinical covariates (Age, T-category, N-category, smoking status, surgery, radiotherapy and chemotherapy). P-values were estimated through Wald-test, log-rank test and trend test as appropriate. Cox model results were reported as Hazard Ratio (HR) with low-risk group treated as baseline and other groups were compared to it. All data was analysed in R-statistical environment v3.2.4 using R packages: survival v2.38-3, survcomp v1.20.0 and MASS v7.3-45.

Model performance was also evaluated. Discrimination tests refer to the model's ability to discriminate between those who have an event and those who do not[11]. This was assessed by the Concordance Index (Harrell's C-Index), which is the probability that for two randomly selected cases the predicted and the actual observed event times have the same order. C-index of 0.5 represents random agreement, and 1.0 perfect agreement between model prediction and reality. Performance was also evaluated using sensitivity, positive predictive value (PPV, precision) and negative predictive value (NPV). For sensitivity, PPV and NPV; patients with censored survival status below 5 years were removed. From the remaining patients, in order to establish ground truth, patients having an event before 5 years were considered as true high-risk and the ones having no event in 5 years were treated as true low-risk.

Calibration tests reflect 'agreement between outcome predictions from the model and the observed outcomes'[11]. Calibration plots were created on training set (resampling: 100 times) depicting estimation of bias-corrected predicted survival probability versus actual (OS or DSS) survival probability values at 5-year. For the validation set calibration, predicted survival probabilities (using Training set fit) were plotted against actual (OS or DSS) survival probabilities at 5-year. Hazard regression (hare) from R package polspline (v1.1.12) was used for the estimation of survival probabilities. Calibration analyses were performed using R package rms (v4.5-0).

Data on comparison of risk factors between the low- and high-risk groups in the molecular biomarkers only model

T category showed statistically significant heterogeneity (p-adjusted=0.015) between low- and high-risk groups when stratified by surgery (**Supplementary Table S7**). Gender, N category, smoking status and HPV status did not. To assess the effect of the heterogeneity of T category across cases in risk groups when stratified by surgery a comparison was undertaken of overall survival outcomes across T categories focussing on the high-risk group (**Supplementary Figure S4**). A significantly higher survival was still seen in the T2 high-risk patients treated with surgery compared to non-surgical treatments (HR=0.37, 95% CI=0.14-1, p=0.042). There were trends in survival between the two modalities in the T1 and T3 groups with widely separated curves, but these did not reach statistical significance.

References

1. Schache AG, Powell NG, Cuschieri KS, et al. HPV-Related Oropharynx Cancer in the United Kingdom: An Evolution in the Understanding of Disease Etiology. *Cancer Res* 2016; **76**(22): 6598-606.
2. Mehanna H, Evans M, Beasley M, et al. Oropharyngeal cancer: United Kingdom National Multidisciplinary Guidelines. *J Laryngol Otol* 2016; **130**(S2): S90-S6.
3. Sobin LH, Gospodarowicz MK, Wittekind C. TNM classification of malignant tumours: John Wiley & Sons; 2011.
4. Amin MB, Greene FL, Edge SB, et al. The Eighth Edition AJCC Cancer Staging Manual: Continuing to build a bridge from a population-based to a more "personalized" approach to cancer staging. *CA Cancer J Clin* 2017; **67**(2): 93-9.
5. Ilyas M, Grabsch H, Ellis IO, et al. Guidelines and considerations for conducting experiments using tissue microarrays. *Histopathology* 2013; **62**(6): 827-39.
6. Jordan RC, Lingen MW, Perez-Ordonez B, et al. Validation of methods for oropharyngeal cancer HPV status determination in US cooperative group trials. *Am J Surg Pathol* 2012; **36**(7): 945-54.
7. Ward MJ, Thirdborough SM, Mellows T, et al. Tumour-infiltrating lymphocytes predict for outcome in HPV-positive oropharyngeal cancer. *Br J Cancer* 2014; **110**(2): 489-500.
8. Cuzick J, Dowsett M, Pineda S, et al. Prognostic value of a combined estrogen receptor, progesterone receptor, Ki-67, and human epidermal growth factor receptor 2 immunohistochemical score and comparison with the Genomic Health recurrence score in early breast cancer. *J Clin Oncol* 2011; **29**(32): 4273-8.
9. Ang KK, Harris J, Wheeler R, et al. Human papillomavirus and survival of patients with oropharyngeal cancer. *N Engl J Med* 2010; **363**(1): 24-35.
10. Singhi AD, Westra WH. Comparison of human papillomavirus in situ hybridization and p16 immunohistochemistry in the detection of human papillomavirus-associated head and neck cancer based on a prospective clinical experience. *Cancer* 2010; **116**(9): 2166-73.

Supplementary Tables

Supplementary Table S1.

Materials and methods. [1]Verified diagnostic test performed in a pathology laboratory with Clinical Pathology Accreditation (CPA (UK) Ltd). The remaining products were optimised for the study. [2]INFORM HPV III Family 16 Probe B detects HPV-16, -18, -31, -33, -35, -39, -45, -51, -52, -56, -58, -66. Benchmark = Ventana Benchmark Ultra (Ventana Medical Systems Inc). BOND = BOND-MAX (Leica Biosystems). DAB = 3,3'-diaminobenzidine. IVD = In Vitro Diagnostic Device with CE marking. MCC1 Ventana Benchmark Ultra automated retrieval protocol. NA = not applicable. rho value - correlation between scorers. RTU = Ready to Use (pre-diluted). RUO = Research Use Only. SCC1 Ventana Benchmark Ultra automated retrieval protocol.

Product	Clone	Species	Licencing	Manufacturer	Dilution	Method	Retrieval	Detection	No. of scorers	Correlation between pathologists min-max rho values
BCL-2[1]	Bcl-2/ 100/D5	Mouse	IVD	Leica	1 in 10	Benchmark	SCC1	Ultraview	4	0.89-0.97
COX-2	SP21	Rabbit	IVD	Ventana/ Cell Marque	RTU	Benchmark	SCC1	Ultraview	4	0.58-0.72
CyclinD1[1]	SP4	Rabbit	RUO	Abcam	1 in 100	Benchmark	SCC1	Ultraview	4	0.9-0.96
EGFR external	EGFR.113	Mouse	IVD	Leica	1 in 100	Benchmark	SCC1	Optiview	3	0.85-0.89
HIF1α	EP1215Y	Rabbit	RUO	Abcam	1 in 400	Benchmark	MCC1	Optiview	3	0.65-0.82
p16[1]	E6H4	Mouse	IVD	Roche mtm labs	RTU	Benchmark	SCC1	Ultraview	4	0.78-0.88
PLK1	MJS1	Mouse	IVD	Leica	1 in 100	BOND	Heat pH6	Novolink	4	0.76-0.83
Survivin	EP2880Y	Rabbit	RUO	Abcam	1 in 750	Manual	Heat pH6	DAB	4	0.76-0.84
INFORM HPV III Family 16 Probe (B)[1, 2]	NA	NA	IVD	Roche Ventana	RTU	Benchmark	NA	ISH/VIEW BLUE PLUS	4	-

Supplementary Table S2.

Treatment details.

	Number of patients (n=985)	%
**Overall Treatment**		
Surgery alone	41	4.16
Surgery + adjuvant radiotherapy	240	24.37
Surgery + adjuvant chemoradiotherapy	144	14.62
Surgery, other treatment not known	14	1.42
Surgery overall	439	44.57
**Radiotherapy regimens**		
68-70 Gy in 34-35 fractions	96	9.75
60-66 Gy in 30 fractions	407	41.32
55 Gy in 20 fractions	182	18.48
Other	63	6.4
Missing information	120	12.18
**Chemotherapy regimen**		
Concomitant platinum	270	27.41
Neoadjuvant platinum + docetaxel combination followed by concomitant platinum	140	14.21
Other/unknown	81	8.22

Supplementary Table S3.

Overall survival difference between missing data group and complete data group by biomarker, suggesting no survival differences between the samples with complete molecular data versus the samples with missing data. *n* represents number of samples including NA and non-NA values for the molecular markers.

	HR	95% CI (Lower)	95% CI (Upper)	P	n
BCL2	0.989256023	0.664965506	1.471696609	0.957492826	833
COX2	0.956736883	0.634194586	1.44331958	0.833028384	833
CyclinD1	0.917870377	0.612824115	1.374759917	0.677569023	833
EGFRext	1.03596752	0.705023411	1.522259665	0.857189148	833
HIF1alpha	0.947779501	0.648701765	1.384744162	0.78158709	833
p16	0.953431061	0.644966465	1.409423338	0.811007526	833
PLK1	1.005833259	0.714092876	1.41676325	0.973452673	833
Survivin	1.045709967	0.715724823	1.527834858	0.817278512	833
HR-HPV ISH	0.993742066	0.658739173	1.499111234	0.976126482	833
TILS	0.812390471	0.600596367	1.098871577	0.177599045	833

Supplementary Table S4.

Mean, median and distribution scores of each biomarker

	Min.	1st Quartile	Median	Mean	3rd Quartile	Max.
Survivin	0	40	62.5	66.52	90	192.5
PLK1	0	15	27.5	35.57	47.5	180
p16	0	0	220	158.7	280	300
HIF1alpha	0	0	7.5	27.96	31.25	280
EGFRext	0	76.67	135	137.8	195.8	300
CyclinD1	0	26.67	99.58	126.5	225	300
COX2	0	100	146.1	147.2	190.8	300
BCL2	0	0	8.33	62.64	100	300

Supplementary Table S5.

Univariable associations of all markers and clinical covariates with overall survival. coef = fitted coefficients (β) of the model. Q is adjusted P-value using Benjamini & Hochberg method. TILS 1 = low (baseline), TILS 2 = moderate, TILS 3 = high. Smoking 0 = never (baseline), 1 = previous, 2 = current. Age (Age.at.Diagnosis) = 1 (>= 50) compared to Age <50 (baseline).

	Coefficient	95% confidence interval (lower)	95% confidence interval (upper)	P	n	Q
BCL2	-0.31657	-0.52514	-0.10800	0.0029311	464	0.00653862
COX2	-0.16834	-0.33675	0.00008	0.05010227	466	0.08546858
CyclinD1	0.60796	0.44038	0.77554	1.16E-12	464	1.68E-11
EGFRext	0.29339	0.12942	0.45736	0.00045329	460	0.00119504
HIF1alpha	-0.12571	-0.31656	0.06513	0.19667864	454	0.27160384
factor(p16)1	-1.48743	-1.84660	-1.12825	4.79E-16	458	1.39E-14
PLK1	-0.09459	-0.27507	0.08590	0.30433662	449	0.36774008
Survivin	-0.10048	-0.27272	0.07177	0.25289547	458	0.31886821
factor(HPVISHHR)1	-1.33842	-1.86053	-0.81631	5.05E-07	468	2.09E-06
factor(TILS)2	-0.97967	-1.37906	-0.58028	1.53E-06	370	5.54E-06
factor(TILS)3	-1.82965	-2.41381	-1.24550	8.31E-10	370	6.02E-09
factor(T)2	0.34784	-0.24366	0.93935	0.24907944	515	0.31886821
factor(T)3	1.21281	0.63466	1.79097	3.93E-05	515	0.00012668
factor(T)4	1.61698	1.06386	2.17010	1.01E-08	515	5.83E-08
factor(N)1	-0.19808	-0.71483	0.31867	0.45248327	522	0.48600055
factor(N)2	-0.10025	-0.80094	0.60044	0.77916103	522	0.77916103
factor(N)2A	-1.12553	-1.98997	-0.26108	0.01071319	522	0.02071218
factor(N)2B	-0.15731	-0.59571	0.28108	0.48185795	522	0.49906717
factor(N)2C	0.47826	-0.06849	1.02501	0.08644463	522	0.13194181
factor(N)3	0.30506	-0.37022	0.98034	0.37593107	522	0.43608004
factor(N8)2	0.69221	0.24942	1.13501	0.00218423	452	0.00527854
factor(N8)3	-1.50647	-1.96141	-1.05154	8.57E-11	452	8.29E-10
factor(N8)4	-0.38946	-0.95388	0.17496	0.17624386	452	0.2555536
factor(Smoking.status)1	0.51347	-0.02811	1.05504	0.06313504	464	0.10171756
factor(Smoking.status)2	1.31397	0.82097	1.80696	1.75E-07	464	8.47E-07
factor(RT)1	-0.70890	-1.27757	-0.14023	0.014554	524	0.02637913
factor(CT)1	0.14747	-0.21581	0.51076	0.42624366	482	0.47542562
factor(Surgery)1	-0.64516	-0.97597	-0.31434	0.0001322	528	0.00038338
factor(Age.at.Diagnosis)1	0.64716	0.19609	1.09824	0.00492382	527	0.01019933

Supplementary Table S6.

Multivariable prognostic model of overall survival based on molecular biomarkers using the training cohort (n = 266, number of events = 69). Significance codes: *** : P<0.001, ** : P < 0.01, * : P<0.05, . : P < 0.1. coef = fitted coefficients of the model. A negative value denotes improvement in survival. exp(coef) = exponent of the coefficients, hazard ratio. se(coef) = standard error of coefficients. z = Wald statistics' z-score. Pr = Probability value. TILS 1 = low (baseline), TILS 2= moderate TILS. TILS3 = high TILS.

	coef	exp(coef)	se(coef)	z	Pr(>|z|)	Significance
factor(p16)1	-0.9911	0.3712	0.3092	-3.205	0.00135	**
Survivin	0.1678	1.1827	0.1255	1.337	0.18112	
factor(HPV ISH)1	-1.4105	0.244	0.5118	-2.756	0.00586	**
factor(TILS)2	-0.6547	0.5196	0.2703	-2.422	0.01544	*
factor(TILS)3	-1.301	0.2723	0.409	-3.181	0.00147	**

Supplementary Table S7.

Comparison of surgery versus no-surgery in predicted low and high-risk groups of validation cohort. HPV 0 = No, 1 = Yes. Smoking 0 = None, 1 = Previous, 2 = Current. q = adjusted p values of Fisher's exact test.

Risk	Surgery	Gender	T	N	Smoking	HPVISH
Low	No	F = 7<br>M = 31	1 = 7<br>2 = 12<br>3 = 7<br>4 = 12	0 = 5<br>1 = 5<br>2 = 1<br>2A = 8<br>2B = 14<br>2C = 4<br>3 = 1	0 = 14<br>1 = 14<br>2 = 10	0 = 8<br>1 = 30
Low	Yes	F = 9<br>M = 54	1 = 13<br>2 = 38<br>3 = 10<br>4 = 2	0 = 7<br>1 = 5<br>2 = 2<br>2A = 17<br>2B = 28<br>2C =1<br>3 = 3	0 = 27<br>1 = 23<br>2 = 13	0 = 12<br>1 = 51
		q = 0.802	q = 0.002	q = 0.802	q = 0.802	q = 0.802
High	No	F = 20<br>M = 35	1 = 4<br>2 = 14<br>3 = 16<br>4 = 21	0 = 23<br>1 = 7<br>2 = 3<br>2A = 5<br>2B = 8<br>2C = 5<br>3 = 4	0 = 5<br>1 = 11<br>2 = 39	0 = 55<br>1 = NA
High	Yes	F = 18<br>M = 47	1 = 14<br>2 = 25<br>3 = 17<br>4 = 9	0 = 25<br>1 = 7<br>2 = 1<br>2A = 7<br>2B = 20<br>2C = 2<br>3 = 3	0 = 9<br>1 = 22<br>2 = 34	0 = 65<br>1 = NA
		q = 0.372	q = 0.015	q = 0.372	q = 0.257	q = NA

Supplementary Table S8.

Multivariable prognostic model of overall survival based on all cofactors (clinical and molecular biomarkers) using the training cohort (n= 266, number of events= 69). Significance codes: *** : P<0.001, ** : P< 0.01, * : P<0.05, . : P<0.1. coef = fitted coefficients of the model. A negative value denotes improvement in survival. exp(coef) = exponent of the coefficients, hazard ratio. se(coef) = standard error of coefficients. z = Wald statistics' z-score. Pr = Probability value. TILS 1 = low (baseline), TILS 2= moderate TILS. TILS3 = high TILS. Smoking 0 = never (baseline), 1 = previous, 2 = current.

	coef	exp(coef)	se(coef)	z	Pr(>|z|)	Significance
BCL2	-0.341646	0.7106	0.147313	-2.319	0.020385	*
CyclinD1	0.452352	1.572006	0.177261	2.552	0.010714	*
HIF1alpha	-0.337002	0.713908	0.190248	-1.771	0.076497	.
PLK1	-0.29237	0.746492	0.166797	-1.753	0.079627	.
Survivin	0.357149	1.429248	0.165019	2.164	0.030442	*
HPVISHHR1	-1.679552	0.186458	0.522342	-3.215	0.001303	**
TILS2	-0.863822	0.421548	0.295914	-2.919	0.00351	**
TILS3	-0.873856	0.417339	0.434696	-2.01	0.044403	*
T2	1.510202	4.527646	0.77367	1.952	0.050939	.
T3	2.307621	10.050487	0.764721	3.018	0.002548	**
T4	2.587341	13.294378	0.753243	3.435	0.000593	***
N1	-0.003545	0.996461	0.420488	-0.008	0.993273	
N2	0.450984	1.569857	0.585452	0.77	0.441111	
N2A	-0.409848	0.663751	0.771255	-0.531	0.595139	
N2B	0.934799	2.546701	0.363162	2.574	0.010051	*
N2C	1.669358	5.308757	0.534724	3.122	0.001797	**
N3	0.358048	1.430534	0.712843	0.502	0.615469	
Smoking.status1	0.163625	1.177773	0.445748	0.367	0.71356	
Smoking.status2	0.836363	2.307958	0.422608	1.979	0.04781	*

Supplementary Table S9.

Multivariable prognostic model of overall survival based on clinical cofactors only, with TNM8, using the training cohort (n= 266, number of events= 69). Significance codes: *** : P<0.001, ** : P < 0.01, * : P<0.05, . : P < 0.1. coef = fitted coefficients of the model. A negative value denotes improvement in survival. exp(coef) = exponent of the coefficients, hazard ratio. se(coef) = standard error of coefficients. z = Wald statistics' z-score. Pr = Probability value. Smoking 0 = never (baseline), 1 = previous, 2 = current.

	coef	exp(coef)	se(coef)	z	Pr(>|z|)	Significance
T2	1.3948	4.0342	0.7524	1.854	0.06376	.
T3	2.1378	8.4805	0.7411	2.885	0.00392	**
T4	2.4248	11.3005	0.7424	3.266	0.00109	**
N82	0.9015	2.4633	0.338	2.667	0.00765	**
N83	-1.0355	0.3551	0.364	-2.845	0.00444	**
N84	-0.4977	0.6079	0.4432	-1.123	0.26145	
Smoking.status1	0.3455	1.4126	0.4112	0.84	0.40083	
Smoking.status2	1.0427	2.8368	0.4024	2.591	0.00957	**

Supplementary Table S10.

Multivariable prognostic model of overall survival based on clinical cofactors only, with TNM7, using the training cohort (n= 266, number of events= 69). Significance codes: *** : P<0.001, ** : P < 0.01, * : P<0.05, . : P < 0.1. coef = fitted coefficients of the model. A negative value denotes improvement in survival. exp(coef) = exponent of the coefficients, hazard ratio. se(coef) = standard error of coefficients. z = Wald statistics' z-score. Pr = Probability value. Smoking 0 = never (baseline), 1 = previous, 2 = current.

	coef	exp(coef)	se(coef)	z	Pr(>|z|)	Significance
T2	1.3628	3.907	0.7502	1.817	0.069292	.
T3	2.3557	10.5457	0.7375	3.194	0.001403	**
T4	2.9132	18.416	0.7333	3.973	7.10E-05	***
Smoking.status1	0.418	1.5189	0.4063	1.029	0.303542	
Smoking.status2	1.3475	3.8477	0.3697	3.645	0.000268	***

Supplementary Table S11.

Sensitivity comparison of models using complete cases set versus imputed Imputation was performed on molecular biomarkers only and not on patient's clinical covariates.

Model	Complete cases validation on results	Imputed dataset validation result pmmtype=1 boot method="simple"	Imputed dataset Validation results pmmtype=1 boot.method= "approximate bayesian"	Imputed dataset validation results pmmtype=2 boot.method= "simple"	Imputed dataset validation results pmmtype=2 boot.method= "approximate bayesian"
Molecular Biomarkers only	C-index=0.73 Sensitivity=0.80 PPV=0.63 NPV=0.75	C-index=0.75 Sensitivity=0.70 PPV=0.63 NPV=0.66	C-index=0.75 Sensitivity=0.61 PPV=0.62 NPV=0.61	C-index=0.76 Sensitivity=0.70 PPV=0.63 NPV=0.66	C-index=0.70 Sensitivity=0.76 PPV=0.62 NPV=0.69
Composite	C-index=0.73 Sensitivity=0.77 PPV=0.64 NPV=0.74	C-index=0.73 Sensitivity=0.77 PPV=0.65 NPV=0.72	C-index=0.74 Sensitivity=0.75 PPV=0.65 NPV=0.71	C-index=0.73 Sensitivity=0.75 PPV=0.66 NPV=0.71	C-index=0.72 Sensitivity=0.74 PPV=0.65 NPV=0.7
Clinical only	C-index=0.73 Sensitivity=0.74 PPV=0.65 NPV=0.72	NA	NA	NA	NA

Supplementary Figure S1.

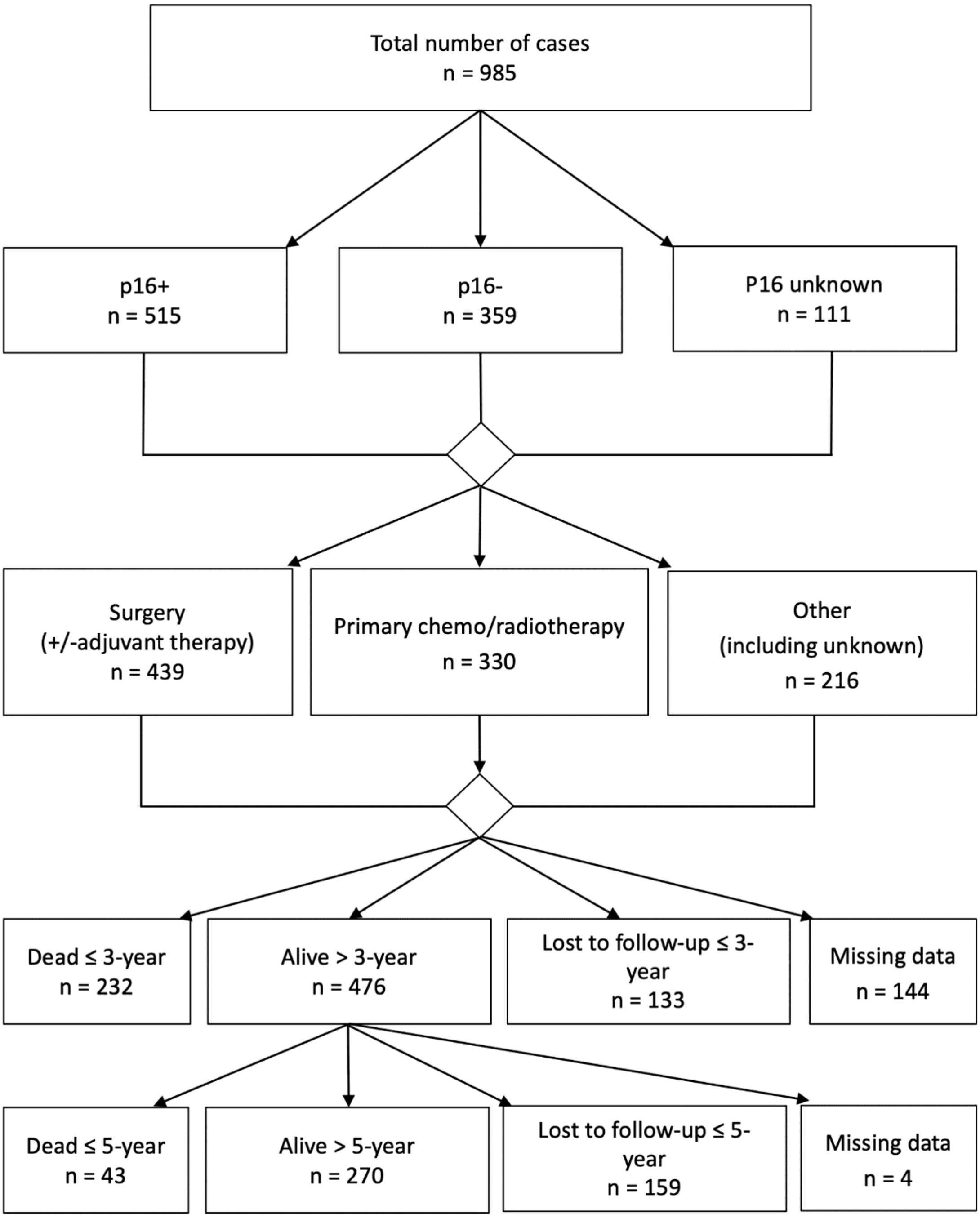

REMARK equivalent patient flow diagram describing breakdown of patients across p16, treatment and survival groups.

Supplementary Figure S2.

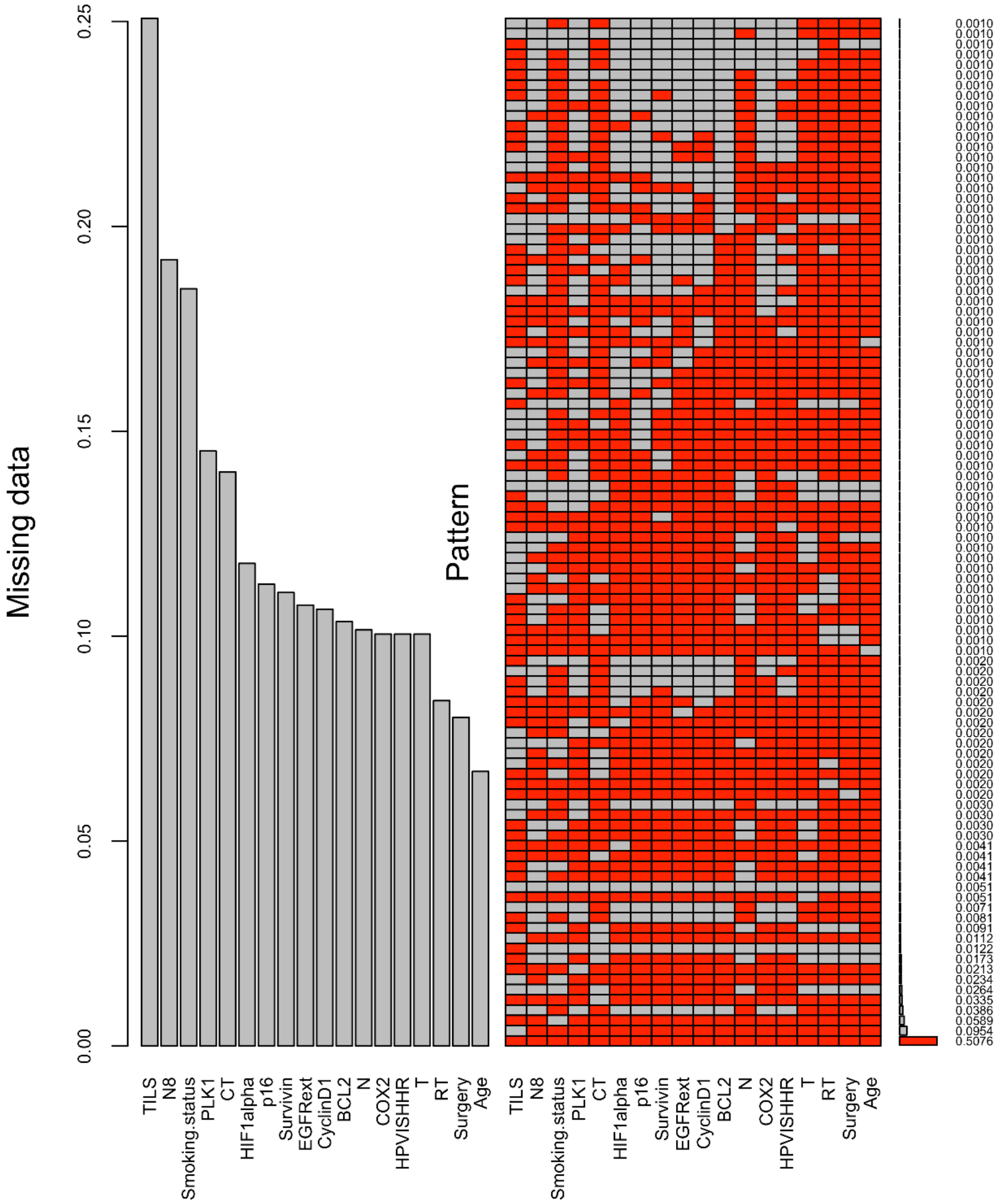

Barchart (left) shows proportion of missing values across various molecular and clinical covariates. Heatmap (right) shows combinatorial prevalence of missing values. Red indicates presence and grey indicates absence.

Supplementary Figure S3.

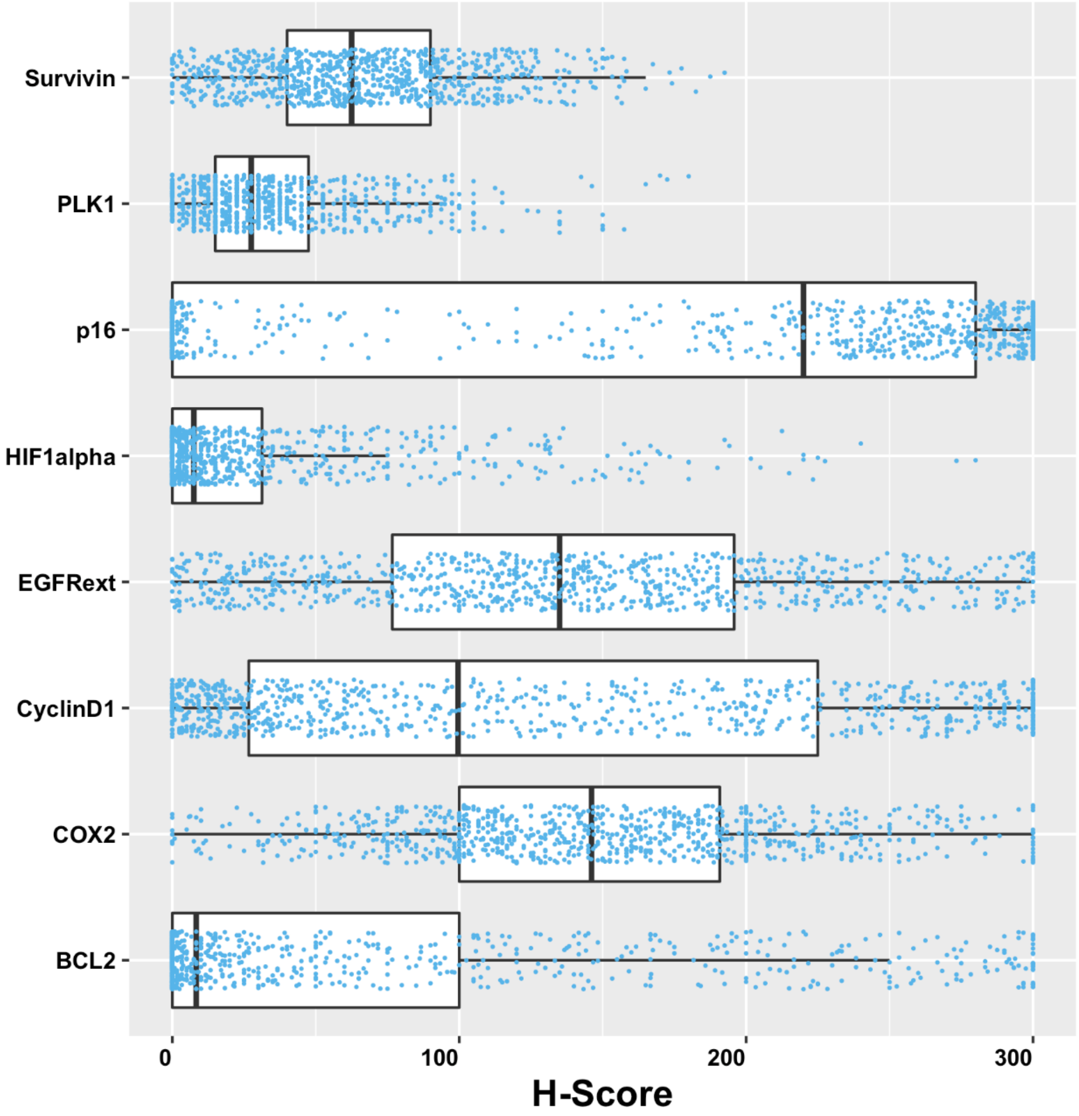

H-score distribution for the biomarkers

Supplementary Figure S4.

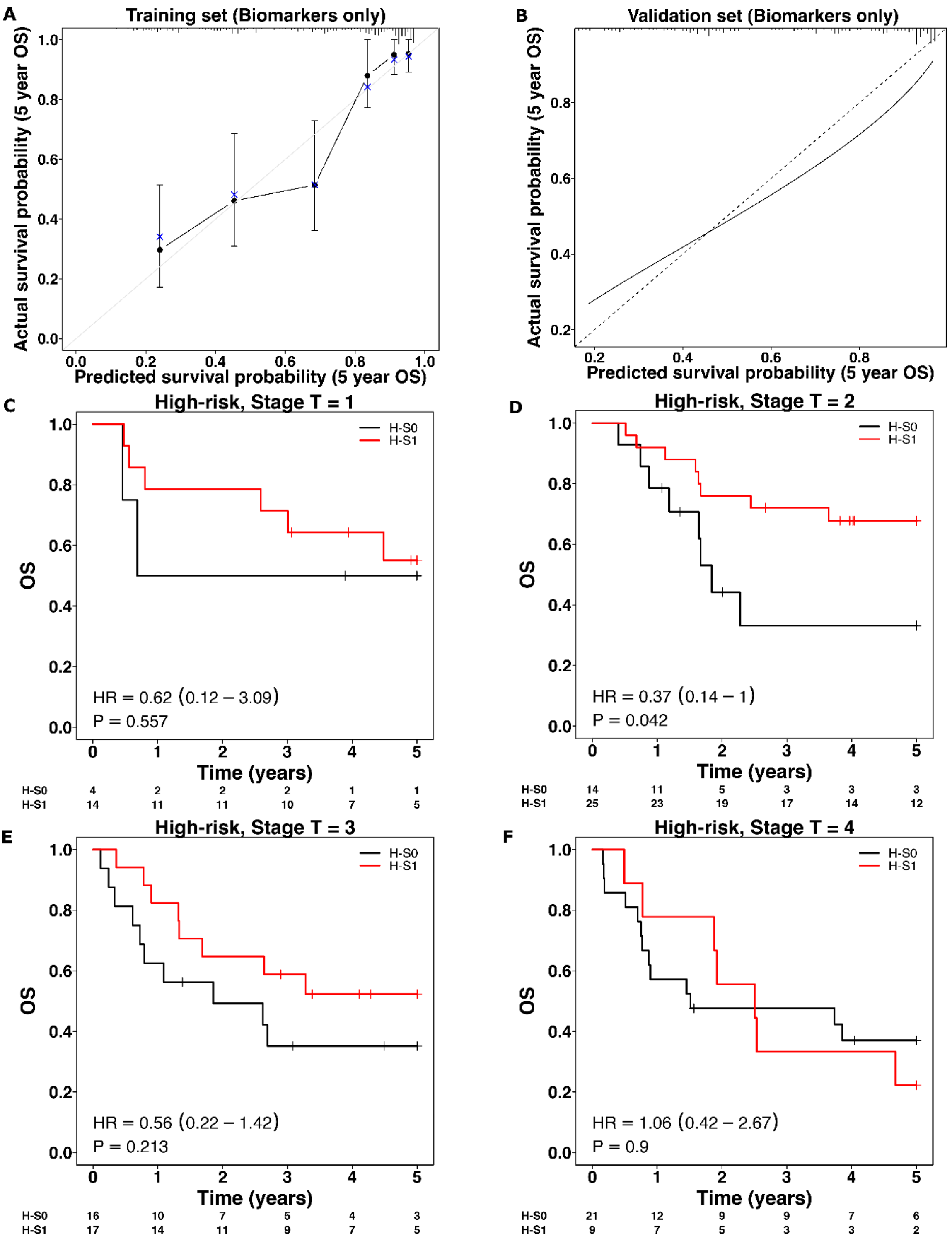

(A, B) Calibration plots of the training and validation cohorts for the biomarker only and the composite classifiers. (C-F) Comparison of overall survival of surgery versus no surgery groups in high-risk groups across T categories in the validation cohort of the molecular biomarkers only model.

Supplementary Figure S5.

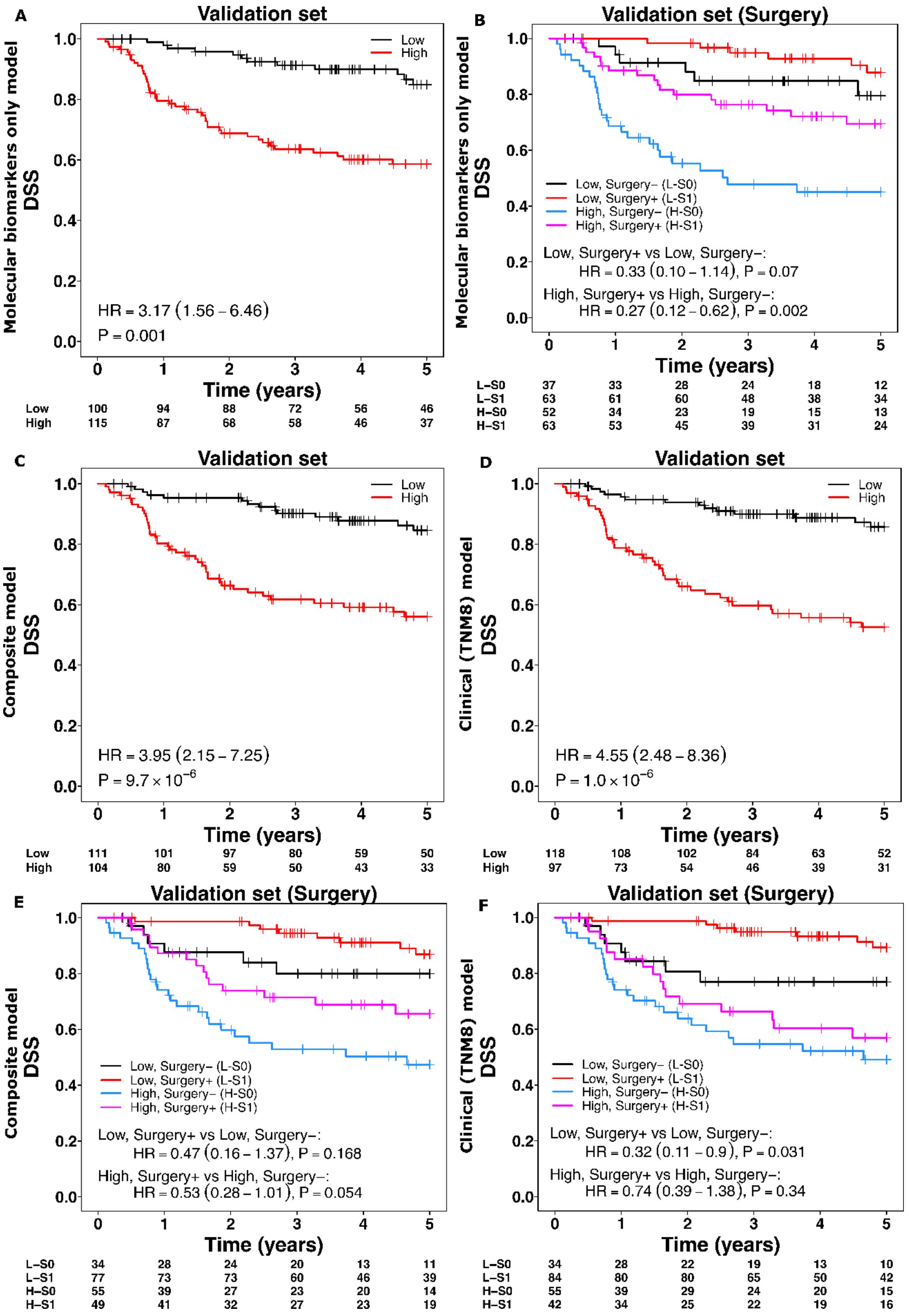

(A) Molecular biomarkers only (trained with OS) in validation set patients'

disease specific survival (DSS). (B) Prognostic evaluation of model in (A) stratified by Surgery. (C, D) Prognostic evaluation of composite (C) and clinical (TNM8) only (D) models (trained with OS) in validation set patients' disease specific survival (DSS). (E, F) Prognostic evaluation of models in (C, D) stratified by Surgery. [Colour key A, C, D: Red = high-risk, Black= low-risk group; B, E, F: Red = low-risk surgery, black = low-risk no surgery; pink = high-risk surgery; blue = high-risk no surgery groups].

Supplementary Figure S6.

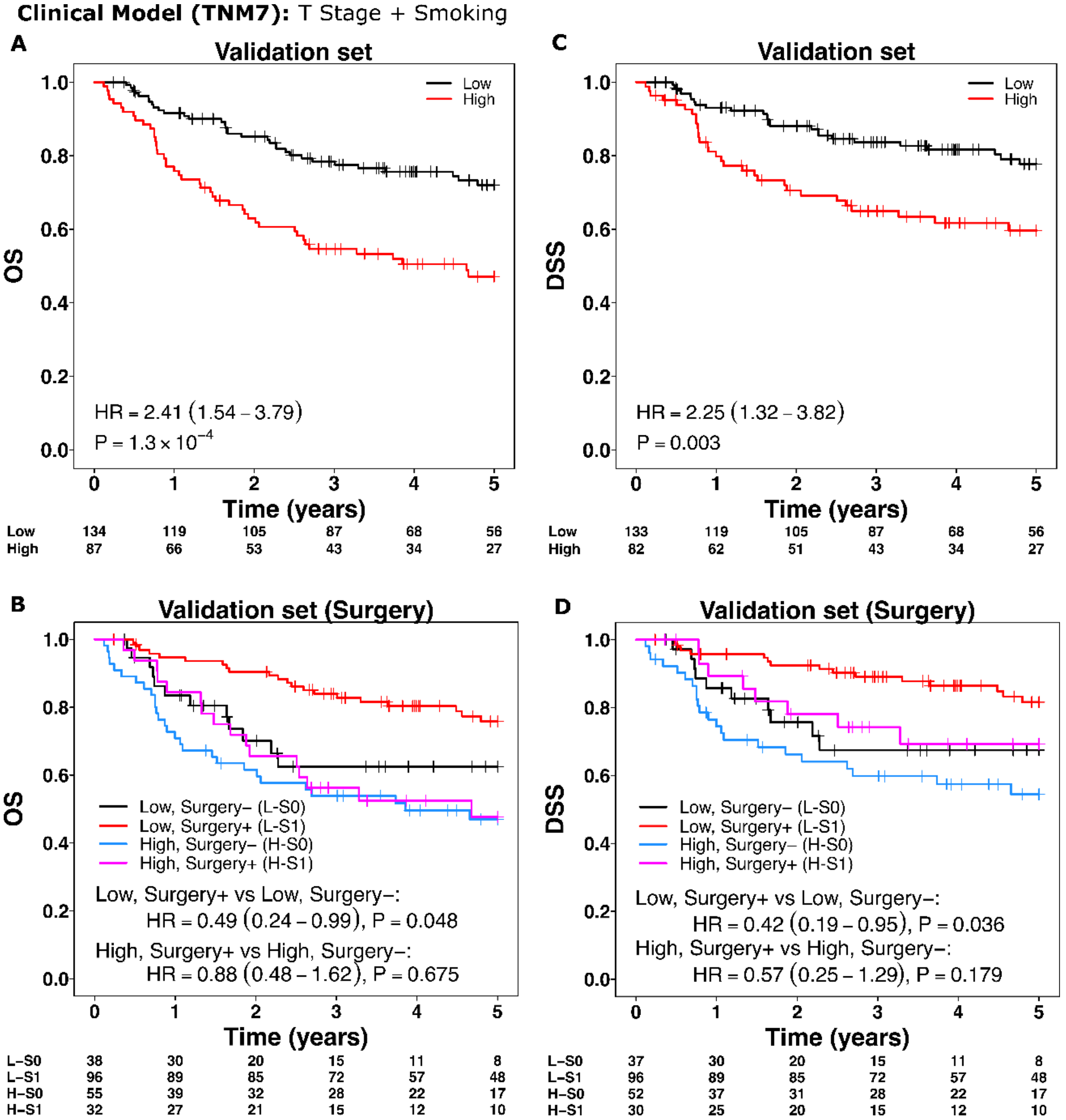

Validation of multivariable survival model trained on clinical factors only (TNM 7, age and smoking status, using backward elimination with Akaike Information Criterion) for overall survival. Validation was performed on OS and DSS [Colour key A, C: Red = high-risk, Black=low-risk group; B, D: Red=low-risk surgery, black = low-risk no surgery; pink = high-risk surgery; blue= high-risk no surgery groups].
